# Electronic trap detection with carrier-resolved photo-Hall effect

**DOI:** 10.1126/sciadv.adz0460

**Published:** 2026-01-01

**Authors:** Oki Gunawan, Chaeyoun Kim, Bonfilio Nainggolan, Minyeul Lee, Jonghwa Shin, Dong Suk Kim, Yimhyun Jo, Minjin Kim, Julie Euvrard, Douglas Bishop, Frank Libsch, Teodor Todorov, Yunna Kim, Byungha Shin

**Affiliations:** ^1^ IBM T. J. Watson Research Center, Yorktown Heights, NY 10598, USA.; ^2^ Dept. of Materials Science and Engineering, Korea Advanced Institute of Science and Technology, Daejeon 34141, Republic of Korea.; ^3^ Dept. of Physics, Arizona State University, Tempe, AZ 85287, USA.; ^4^ Ulsan Advanced Energy Technology R&D Center, Korea Institute of Energy Research, Ulsan 44776, Republic of Korea.; ^5^ Graduate School of Carbon Neutrality, Ulsan National Institute of Science and Technology, Ulsan 44919, Republic of Korea.; ^6^ Dept. of Physics and Centre for Processable Electronics, Imperial College London, London SW7 2AZ, UK.

## Abstract

Electronic trap states critically affect the performance of semiconductor devices such as transistors, memory devices, and solar cells. Yet, conventional trap measurement techniques often require junction fabrication, which can introduce or alter traps. We present a unique photo-Hall–based method to characterize trap density and energy levels while concurrently extracting charge carrier properties. By analyzing photo-Hall conductivity versus electrical conductivity under varying light intensities and temperatures, we uncover an astonishingly simple hyperbola relationship that reveals detailed charge transport and trap occupation and applied it in silicon and halide perovskite films. This technique substantially expands Hall effect–based measurements by integrating electric, magnetic, photon, and phonon excitations into a single framework and enables unparalleled extraction of charge carrier and trap properties, offering a powerful tool for semiconductor characterization and device optimization.

## Teaser

Discovery of a hyperbola equation in the photo-Hall effect, advancing semiconductor metrology and trap detection.

## INTRODUCTION

The performance of semiconductor devices across a wide range of applications can be affected by the presence of trap states. Traps in semiconductors originate from defects or impurities that capture and release free charge carriers. The effect of the traps can modify or dominate the electrical transport and charge carrier recombination process in many devices, often reducing conductivity or decreasing charge carrier lifetime. Traps can also give rise to hysteresis, noise in electrical screening, or electrical leakage and unwanted power dissipation. The sources of traps include impurities and crystalline defects (*1*) such as point defects, extended defects (dislocations, grain boundaries, and stacking fault), surface states, and interface states. Understanding and controlling these traps is crucial for optimizing the performance of semiconductor devices. The origin and passivation of trap states has been extensively studied in silicon-based materials but continues to be an area of focus in optoelectronic devices such as solar cells and light emitting diodes, as well as in small channel devices where interface states dominate transport. In other material systems, trap states are less explored but equally important. In particular, lead-halide perovskite systems have shown great promise for optoelectronic applications such as solar cells, recently surpassing the record power conversion efficiency of 26.7% in single-junction configurations (*2*–*5*). The material system has shown inherently low trap density, enabling their high performance; however, managing and further reducing residual traps is increasingly critical for further performance improvements (*6*, *7*).

Techniques to detect traps in semiconductors include deep-level transient spectroscopy (DLTS) (*8*, *9*), drive-level capacitance profiling (*10*, *11*), space charge–limited current (*12*, *13*), thermal admittance spectroscopy (*14*, *15*), transient photoluminescence (*16*), time-resolved microwave conductivity (*17*), photo-Hall spectroscopy (*18*), transient (*19*), and constant light photo-Hall effect (*20*). Each technique offers unique advantages tailored to different aspects of trap properties measurement and suffers from some limitations. See the section SE for a summary. In this study, we present a technique based on recently developed carrier-resolved photo-Hall (CRPH) effect (*21*) that addresses the major drawbacks of existing approaches including the need for a *p-n* or Schottky junction, the impact of interfaces associated with such device, and the transient nature of most measurements. This new carrier and trap–resolved photo-Hall (CTRPH) technique also provides access to a rich set of charge carrier parameters of up to 17 charge carrier parameters that can be mapped against varying light intensities, including the electron and hole mobilities, photocarrier and trapped carrier density, recombination lifetimes, and diffusion lengths; four parameters associated with the dominant trap state (density, energy level, and scattering cross sections). See section SC for a complete list and descriptions.

## RESULTS

### The experiment

**Fig. 1.**
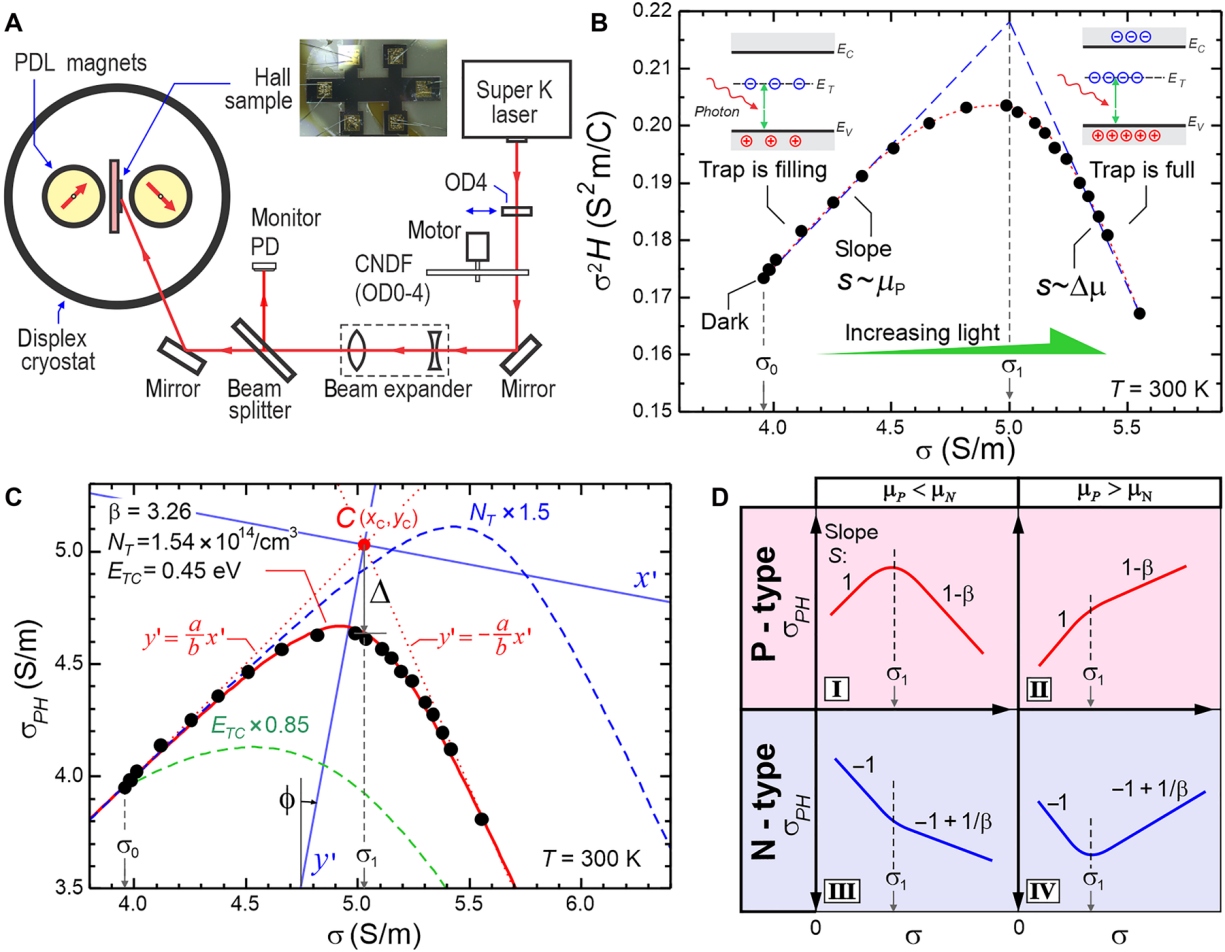
Electronic trap detection with CTRPH technique. (**A**) The experimental setup based on rotating PDL magnet Hall system with cryostat. PD is photodetector, OD is optical density filter, and CNDF is continuous neutral density filter. Inset: the p-type SOI Hall sample. (**B**) The photo-Hall experiment plot of $\sigma^{2}H$ versus σ in a p-type SOI sample with laser light of wavelength λ = 615 nm, maximum intensity *I_L_* = 157 mW/cm^2^, and temperature *T* = 300 K. The dashed curve is only a guide to the eye. (**C**) Hyperbolic fit (red curve) of the data in Fig. 1B. The *x*′-*y*′ is the principal axis of the hyperbola centered at point *C*. The values of fit parameters β, *N*_*T*_, and *E*_*TC*_ are indicated. The impact of varying *N*_*T*_ and *E*_*TC*_ are simulated in the blue and green dashed-curve, respectively. (**D**) Four possible scenarios (marked as quadrant I-IV) of photo-Hall behavior with trapping effect depending on carrier type (P or N) and the relative value of the hole and electron mobility. The dashed lines separate the first and second regime of the curves.

The experimental setup is shown in Fig. 1A. The Hall sample is mounted inside a cryostat with temperature control ranging from 20 to 340 K. The sample is placed between two rotating parallel dipole line (PDL) magnets that generate a strong perpendicular oscillating magnetic field (*22*, *23*) with peak amplitude of *B* ~ 0.5 T. We use a supercontinuum white laser that passes through a monochromator and a set of optics to control light intensity and beam size. To demonstrate our trap detection technique, we use a p-type silicon-on-insulator (SOI) sample on glass with thickness *d* = 5.0 μm. The device is a six-terminal Hall bar (see Fig. 1A, inset). The detailed experimental setup and sample fabrication are described in Materials and Methods. The experiment on p-SOI sample represents a photo-Hall experiment in low-injection regime, and later, we will also present data on perovskite sample that represents a high-injection regime.

The measurement is similar to previously demonstrated CRPH technique (*21*)—In brief, we measure the electrical conductivity σ and Hall coefficient *H* with increasing light intensity and plot $\sigma^{2}H$ versus σ (*21*) as shown in Fig. 1B. In our previous work (*21*), we demonstrated that the minority and majority carrier mobilities can be extracted from the slope $s=d\left(\sigma^{2}H\right)/d\sigma$, which yield the difference between the hole and electron mobility, i.e., $s=\Delta \mu_{Η}=r\left(\mu_{P}-\mu_{N}\right)$, where *r* is the Hall scattering factor and is assumed to be ~1 (*24*). This model applies when the trapping effect is negligible (as in high-quality materials or high light intensity), allowing us to assume that the electron and hole photocarrier density are equal (Δn=Δp). If we extend this model by considering trapping effect, where all the minority carriers are trapped, we show that the slope will yield the majority carrier mobility $\mu_{0}$ i.e. $s∼\mu_{0}$ (see section SA.1 for derivation).

The key insight of the trap detection using CTRPH technique can be seen in the $\sigma^{2}H-\sigma$ trace where there is a bending that demarcates two regimes of the plot with positive and negative slope at low and high light intensity, respectively. If we draw two asymptotic lines as shown in Fig. 1B, we obtain slope of *s* = 440 and −990 cm^2^/Vs, respectively, which is consistent with the known mobility values of hole (the majority carrier) in silicon: $\mu_{P}$ ~500 cm^2^/Vs and the mobility difference $\Delta \mu =\mu_{P}-\mu_{N}$ ~1000 cm^2^/Vs (*1*). We can understand this behavior by considering trapping effect of the minority carrier as shown in Fig. 1B (inset). At low light intensity, the photo-generated minority carriers initially populate the traps, and only free photo-generated majority carriers (Δ*p*) contribute to the conductivity, thus $s∼\mu_{P}$. Once all the traps are filled at a certain light intensity, the photo-generated minority carriers (Δ*n*) will populate the conduction band (CB) and start contributing to the conductivity. In the limit where the photo-generated carrier density Δ*n* and Δ*p* are much higher than the trap density *N*_*T*_, we obtain Δn∼Δp and $s∼\Delta \mu_{H}$ as observed in our earlier study where trapping effects are negligible (*21*, *25*).

### Theoretical model

We now proceed to a more quantitative analysis of the trap properties. To describe the photo-Hall effect in the presence of traps, we model a single-level trap in a P-type material with density *N*_*T*_ and energy *E*_*T*_, which is closer to CB, thus acting as minority carrier trap (see section SA.1). As the light intensity is increased, the electron quasi-Fermi level increases and the trapped (*n*_*T*_) and free (Δ*n*) electron density can be calculated. To simplify our analysis, we define a new quantity: $\sigma_{\mathrm{PH}}=\sigma^{2}H/r\mu_{0}$, which we refer to as “photo-Hall conductivity,” and shares the same dimension as σ. We can show that the $\sigma^{2}H$versus σ plot in the presence of traps can be described by a simple hyperbola equation (see section SA.2 for derivation)

$$\frac{{\sigma_{\mathrm{PH}}}^{\prime 2}}{a^{2}}-\frac{\sigma^{\prime 2}}{b^{2}}=1$$ (1)

where (σ′, σ*_PH_*′) are (σ, σ*_PH_*) expressed in a second coordinate system *x*′-*y*′, which is centered at point *C* and rotated by angle ϕ as shown in Fig. 1C. We have $\phi =\frac{1}{2}{\text{tan}}^{-1}\left(2/\beta -1\right)$, where $\beta =\mu_{N}/\mu_{P}$ is the electron-to-hole mobility ratio. To describe the hyperbola equation parameters, we define another two parameters: $s_{N}=e\mu_{0}\left(p_{0}+N_{T}\right)$ and $s_{E}=e\;g_{T}\mu_{0}\left(1+\beta \right)\;N_{C}\text{exp}\left(-\varepsilon_{\mathrm{TC}}\right)$ where $\varepsilon_{\mathrm{TC}}=E_{TC}/k_{B}T$, with $E_{\text{TC}}=E_{C}-E_{T},E_{C}$ is the CB edge, *e* is the electron’s charge, $p_{0}$ is the majority carrier density in the dark, *N*_*T*_ is the trap density, $g_{T}$ is trap energy level degeneracy, which can be assumed to be equal to 1 for deep level traps (*26*), *N*_C_ is the CB effective density of states, *k*_B_ is the Boltzmann constant, and *T* is the temperature. The parameter $s_{N}$ and $s_{E}$ have clear physical and geometrical meaning: $s_{N}$ depends on *N*_*T*_ and determines the horizontal peak position (σ_1_) of the hyperbola; $s_{E}$ depends on *E_T_* and determines the vertical peak position of the hyperbola or the vertical gap “Δ” from the center point *C* (see Fig. 1C). They are related as $s_{E}=\Delta^{2}/e\beta^{2}\mu_{0}N_{T}$.

The hyperbola has a center point, *C*, which plays a crucial role in this model by automatically providing straightforward solutions for trap density (*N_T_*) and energy level (*E_T_*). The center point *C* has coordinate $x_{C}=s_{N}-s_{E}$ and $y_{C}=s_{N}+\left(\beta -1\right)s_{E}$. We also have *a* and *b* as the hyperbola semimajor and minor axis, respectively, where $a=\sqrt{-K/\lambda_{-}}$ and $b=\sqrt{K/\lambda_{+}}$, with $K=e\mu_{0}\beta^{2}N_{T}s_{E}$ and $\lambda_{\pm }=\left(\beta -2\pm \sqrt{2\beta^{2}-4\beta +4}\right)/2$ as the eigen values of the hyperbola matrix (see section SA.2). We note a special case when β=2, we have ϕ=0, or in other words, the hyperbola *x*′-*y*′ axes are already aligned with the original $\sigma -\sigma_{\mathrm{PH}}$ axes, thus no rotation is needed. From Eq. 1, we can also express $\sigma_{\mathrm{PH}}$ explicitly as a function of σ (see section SA.1)

$$\begin{array}{c} \sigma_{\mathrm{PH}}\left(\sigma \right)=\\ \left[\beta \left(s_{N}+s_{E}\right)+\left(2-\beta \right)\sigma -\beta \sqrt{\;{\left(s_{N}+s_{E}+\sigma \right)}^{2}-4\left(s_{E}\sigma_{0}+s_{N}\sigma \right)}\right]/2 \end{array}$$ (2)

where $\sigma_{0}$ is the conductivity in the dark. Equation 2 can be used for curve-fitting the experimental data to extract the parameters β, $s_{N}$, $s_{E}$ and thus *N*_*T*_ and *E*_*T*_. This approach was applied in Fig. 1C, which also presents the resulting fitting parameters.

We note a few important features of the hyperbolic curve as described by Eq. 2. The slope $d\sigma_{\mathrm{PH}}/d\sigma$ approaches 1 in the dark at low temperature (or $E_{\mathrm{TC}}\gg k_{B}T$) and 1-β at maximum light intensity. The angle ϕ and the asymptotes $y^{\prime }=\pm \left(b/a\right)\;x^{\prime }$ are solely determined by mobility ratio β. The parameters *N*_*T*_ and *E*_*T*_ determine the horizontal and the vertical positions of the inflection (or vertex) point of the hyperbola, respectively. At larger *N*_*T*_, the inflection point occurs at higher light intensity (or conductivity), as shown in the blue dashed curve in Fig. 1C where *N*_*T*_ is increased by 1.5×. This shift is expected as more photo-generated carriers are required to fill the traps. Meanwhile, smaller trap energy *E*_*TC*_ leads to a smoother hyperbolic curve as shown in the green dashed curve in Fig. 1C, where *E_TC_* is reduced to 0.85×. As the impact of the trap on the minority carrier is temperature dependent, shallow traps ($E_{\mathrm{TC}}∼k_{B}T$) produce smoother hyperbolic curves, while deep traps ($E_{\mathrm{TC}}\gg k_{B}T$) result in sharper hyperbolic curves.

Figure 1D summarizes the expected shape of the $\sigma_{\mathrm{PH}}-\sigma$ trace for four possible scenarios (marked as quadrant I to IV) depending on the material types (*P* or *N*) and the relative mobility values ($\mu_{P}<\mu_{N}$ or $\mu_{P}>\mu_{N}$). Our hyperbola model (Eq. 2) applies for all cases, albeit with minor modifications in formula for N-type systems (see section SA.5). This diagram also reveals important characteristics: The slope changes by the mobility ratio of minority to majority carrier, i.e., −β or + 1/β for P-type and N-type, respectively, which implies that significant bending behavior occurs when the mobility ratio is large (e.g., Fig. 1D, quadrant I and IV). This is reasonable because once the trap becomes full and the system enters a second regime, the minority carrier starts contributing to the transport and the change in slope will be significant if the minority carrier mobility is larger than that of the majority. For example, the p-SOI data in Fig. 1C show quadrant I behavior since it is a P-type with β = 3.26.

## DISCUSSION

### CTRPH analysis in the p-SOI sample

We can now apply a complete CTRPH analysis to the p-SOI data. We can use two approaches to extract the trap parameters *N*_*T*_ and *E*_*TC*_ from the photo-Hall experiment. The first technique would require to fit the experimental data ($\sigma_{\mathrm{PH}}-\sigma$) using Eqs. 1 or 2 and extract β, $s_{N}$, and $s_{E}$ as fitting parameters. This curve fitting is illustrated in Fig. 1C, which yields β = 3.26, *N*_*T*_ = 1.54 × 10^14^/cm^3^, and *E*_*TC*_ = 0.45 eV. The β value is close to 3 as expected for silicon (*1*). This approach is however hindered by the need for effective mass that appears in the effective density of states $N_{C}$ (or $N_{V}$) in calculation involving $s_{E}$, which, while known for many materials, is rarely available for new materials.

In the second technique, which is an alternative to curve fitting, we can perform a geometric analysis of the $\sigma_{\mathrm{PH}}-\sigma$ trace to extract β, $N_{T}$ and $E_{\mathrm{TC}}$. β is extracted from the slope of the high light intensity regime with the asymptotic limit of 1-β and calculated using $\beta =1-{\left(d\sigma_{\mathrm{PH}}/d\sigma \right)}_{\infty }$. Note that β is however not required to calculate *N*_*T*_ and E*_TC_*. When $E_{\mathrm{TC}}\gg k_{B}T$, which can be achieved at low temperature, the hyperbola becomes sufficiently sharp, and the trap density can be estimated with a very simple equation (see section SA.3)

$${\tilde{N}}_{T}=\frac{\sigma_{1}-\sigma_{0}}{e\;\mu_{0}}$$ (3)

where ${\tilde{N}}_{T}$ is the estimated trap density calculated from the difference between the inflection (or vertex) point at $\sigma_{1}$ and the dark conductivity $\sigma_{0}$ as shown in Fig. 1C. The difference in conductivity between the point $\sigma_{1}$ and $\sigma_{0}$ is primarily due to free majority carriers (Δp). As all available trap states are filled by the photo-generated electrons near the inflection point of the hyperbola, the Δp at this point is close to the trap density $N_{T}$. A more exact expression of Eq. 3 that contains temperature correction is given in section SA.3.

Next, we can determine the trap energy *E_TC_* by measuring the gap Δ in Fig. 1C and use the relationship (see section SA.4 for derivation)

$$E_{\mathrm{TC}}=k_{B}T\text{ln}\left[e^{2}g_{T}{\mu_{0}}^{2}\beta^{2}\left(\beta +1\right)N_{C}N_{T}/\Delta^{2}\right]$$ (4)

**Fig. 2.**
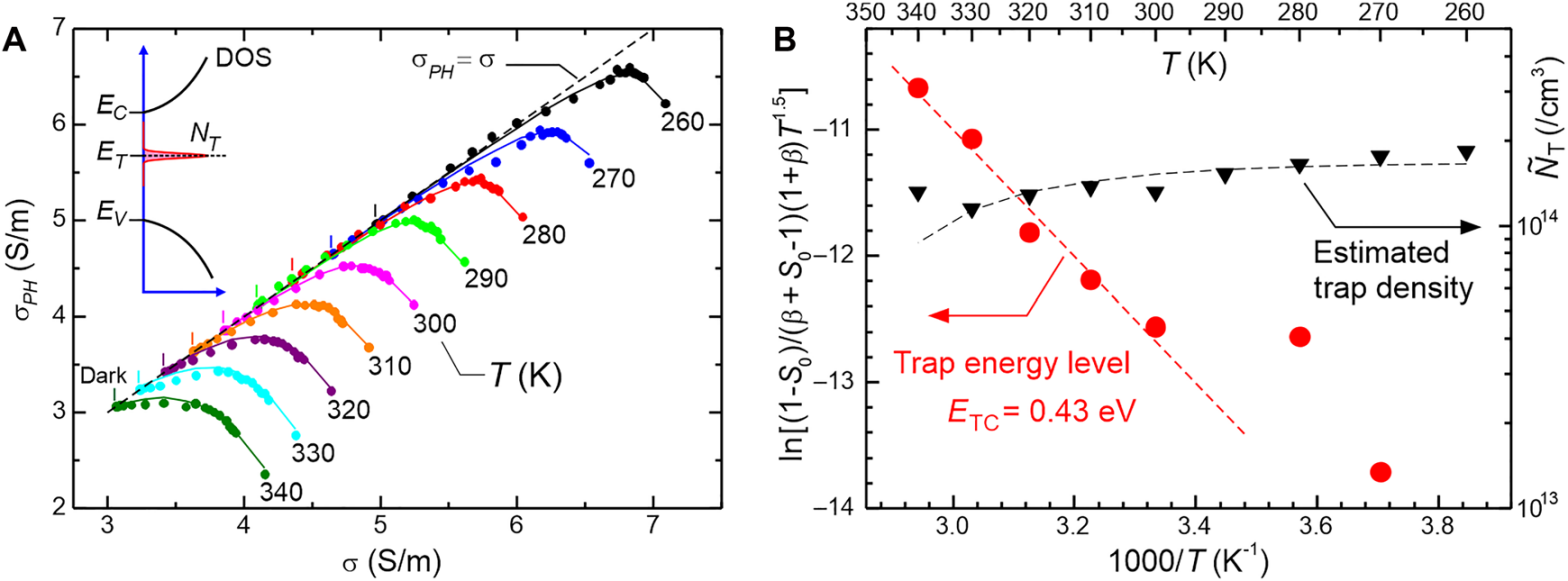
Trap analysis using temperature-dependent CTRPH measurement in the p-SOI sample. (**A**) Photo-Hall data from *T* = 260 to 340 K with laser light of λ = 550 nm and maximum intensity *I_L_* = 165 mW/cm^2^. The dark data points are indicated by short vertical dashes. The dashed line is the $\sigma_{\text{PH}}=\sigma$ asymptotic line. Inset: energy band diagram of the trap model. (**B**) Arrhenius plot of Eq. 5 to extract the trap energy level ($E_{\text{TC}}$) and the estimated trap density (${\tilde{N}}_{T}$) extracted using Eq. 3. Black dashed-curve: curve-fit of temperature-dependent effect of (${\tilde{N}}_{T}$) (see text).

In this example, we have Δ = 0.462 S/m and thus *E*_*TC*_ = 0.46 eV, consistent with direct curve-fitting technique in Fig. 1C. In addition, we can also perform variable temperature photo-Hall measurements, which can provide more accurate access to *E*_*TC*_ without the need for prior knowledge of effective mass or the center point *C*. We demonstrate that the slope $S_{0}={\left[d\sigma_{\mathrm{PH}}/d\sigma \right]}_{0}$ at the dark point is related to $E_{\text{TC}}$ according to (see section SA.4)

$$\text{ln}\left(\frac{1-S_{0}}{\left(\beta +S_{0}-1\right)\left(1+\beta \right)T^{1.5}}\right)=c_{0}-\frac{E_{\text{TC}}}{k_{B}T}$$ (5)

where $c_{0}$ is a constant. Here, we rely on the fact that the slope *S*_0_ drops significantly toward higher temperature as shown in Fig. 2A. We can perform an Arrhenius analysis by plotting the left hand–side quantity versus 1/*T* and extract $E_{\mathrm{TC}}$ from the slope. We apply this variable temperature approach to the photo-Hall measurements of the p-SOI sample at *T* = 260 to 340 K as shown in Fig. 2. We observe that all curves show hyperbolic behavior and become sharper at lower temperature or $E_{\text{TC}}\gg k_{B}T$. The first segment of the curve approaches asymptotic limit $\sigma_{\text{PH}}=\sigma$ toward low temperature as expected from the theory. We obtain trap density *N_T_* = 1.66 × 10^14^ /cm^3^ from the hyperbola peaks and using Eq. 3 at low temperature range (*T* ~ 260 K). This value is close to *N*_*T*_ = 1.54 × 10^14^ /cm^3^ determined from curve-fitting approach in Fig. 1C. We also observe in Fig. 2B that the ${\tilde{N}}_{T}$ values drop slightly at higher temperatures, which is consistent with the theoretical prediction (indicated as black-dashed curve) described in section SA.3. In general, the trap density *N*_*T*_ can be more accurately determined at lower temperature as the hyperbola curve becomes sharper.

Using Eq. 5, we extract $E_{\text{TC}}$ from the slope of the data in Fig. 2B. A linear behavior with respect to 1/*T* is observed at high temperature range (300 to 340 K), the slope yields *E_TC_* = 0.43 eV. At low temperature (*T* < 300 K), the slope *S*_0_ approaches 1, and the left hand–side quantity of Eq. 5, which contains 1-*S*_0_, becomes less accurate and noisier (Fig. 2B). Therefore, the data in this low-temperature range can be excluded. This *E*_*TC*_ value is very close to the value obtained from previous technique using curve-fitting or Δ calculation, i.e., (0.45 eV). Some discrepancy from the first method can be attributed to inaccuracy of point *C* determination (that relies on intersection of two asymptotes) and the value of the effective mass of the SOI sample (which could change under strain). Nevertheless, close and consistent results of $N_{T}$ and $E_{\mathrm{TC}}$ demonstrate the reliability of both approaches. We note that similar trap level of 0.48 eV has been previously observed in SOI samples using DLTS study, and the trap is suspected because of small voids from poorly bonded SOI (*27*).

From Fig. 2, we observe a contrasting trend: *N*_*T*_ and *E*_*TC*_ can generally be determined more accurately at low and high temperatures, respectively. At low temperatures, the hyperbola becomes sharper, allowing its peak position (σ_1_)—used to calculate *N*_*T*_ in Eq. 3—to be determined with higher precision. In contrast, determining *E*_*TC*_ relies on significant variation in the slope *S*_0_, which occurs at higher temperatures. Thus *E*_*TC*_ can be more accurately determined at higher temperature range.

To complete the analysis of the SOI sample, we extend our CTRPH analysis in similar fashion with our previous work (*21*), but now incorporating the presence of traps (see section SB). This approach enables us to fully resolve the concentration of electrons in the trap (*n_T_*), electron photocarrier density (Δ*n*), and hole photocarrier density (Δ*p*) using $n_{T}=\left[\left(\beta -1\right)\;\sigma +\sigma_{\mathrm{PH}}-{\beta \sigma }_{0}\right]/e\mu_{P}\beta$, $\Delta n=\left(\sigma -\sigma_{\mathrm{PH}}\right)/e\mu_{P}\beta \left(\beta +1\right)$, and $\Delta p=\Delta n+n_{T}$ (see section SA.1). Consequently, we can now calculate up to 17 × *N* charge carrier parameters, where *N* represents the number of light intensity settings. A complete list is provided in section SC. The electron and hole charge carrier parameters include photo carrier densities (Δ*n* and Δ*p*), occupied trap density (*n_T_*), mobility ($\mu_{N}$ and $\mu_{P}$), recombination lifetime ($\tau_{N}$ and $\tau_{P}$), diffusion coefficients (*D_N_*, *D_P_*, and *D_A_*), and diffusion lengths (*L_D,N_*, *L_D,P_*, and *L_D,A_*), where the subscript *N*, *P*, and *A* means electron, hole, and ambipolar quantity, respectively. Given the carrier density information, we can also calculate quasi-Fermi levels (*QF_N_* and *QF_P_*), their splitting (*QFLS*) and its ideality factor (η) as suggested in (*20*). In addition, we can extract four trap parameters, including trap density *N*_*T*_, trap energy *E*_*T*_, and recombination scattering cross sections ($\sigma_{P}$ and $\sigma_{N}$). The complete extraction of these parameters, along with relevant plots for the p-SOI sample, is presented in section SD.2.

We have also demonstrated CTRPH analysis in an N-type silicon sample as detailed in section SD.3. The data exhibit broad hyperbola or quadrant III (Fig. 1D) behavior in. From the second segment, we obtain the slope S=−1+1/β = 0.691, which yield β = 3.24 and thus hole (minority) mobility of $\mu_{P}=\mu_{Ν}/\beta =526\;\;{\text{cm}}^{2}/\text{Vs}$. We note that, again, both mobilities are consistent with the known values in silicon of $\mu_{P}$ ~ 500 and $\mu_{N}$ ~ 1500 cm^2^/Vs (*1*), similar to the results from the p-SOI sample. From the inflection point, we obtain trap density $N_{T}$ = 2.5 × 10^12^/cm^3^. For the trap energy level, because of weak bending near the inflection point, we cannot determine the parameter Δ accurately. However we can establish its upper limit, i.e., Δ < 9.4 × 10^−4^ S/m and thus the upper bound of the trap energy using Eq. 4, i.e., $E_{\mathrm{TV}}$ < 0.55 eV. The full set of CTRPH analysis plots, including carrier density, quasi-Fermi levels, mobility, lifetime, diffusion lengths, and ideality factors, are presented in fig. S6.

### CTRPH analysis in perovskite

**Fig. 3.**
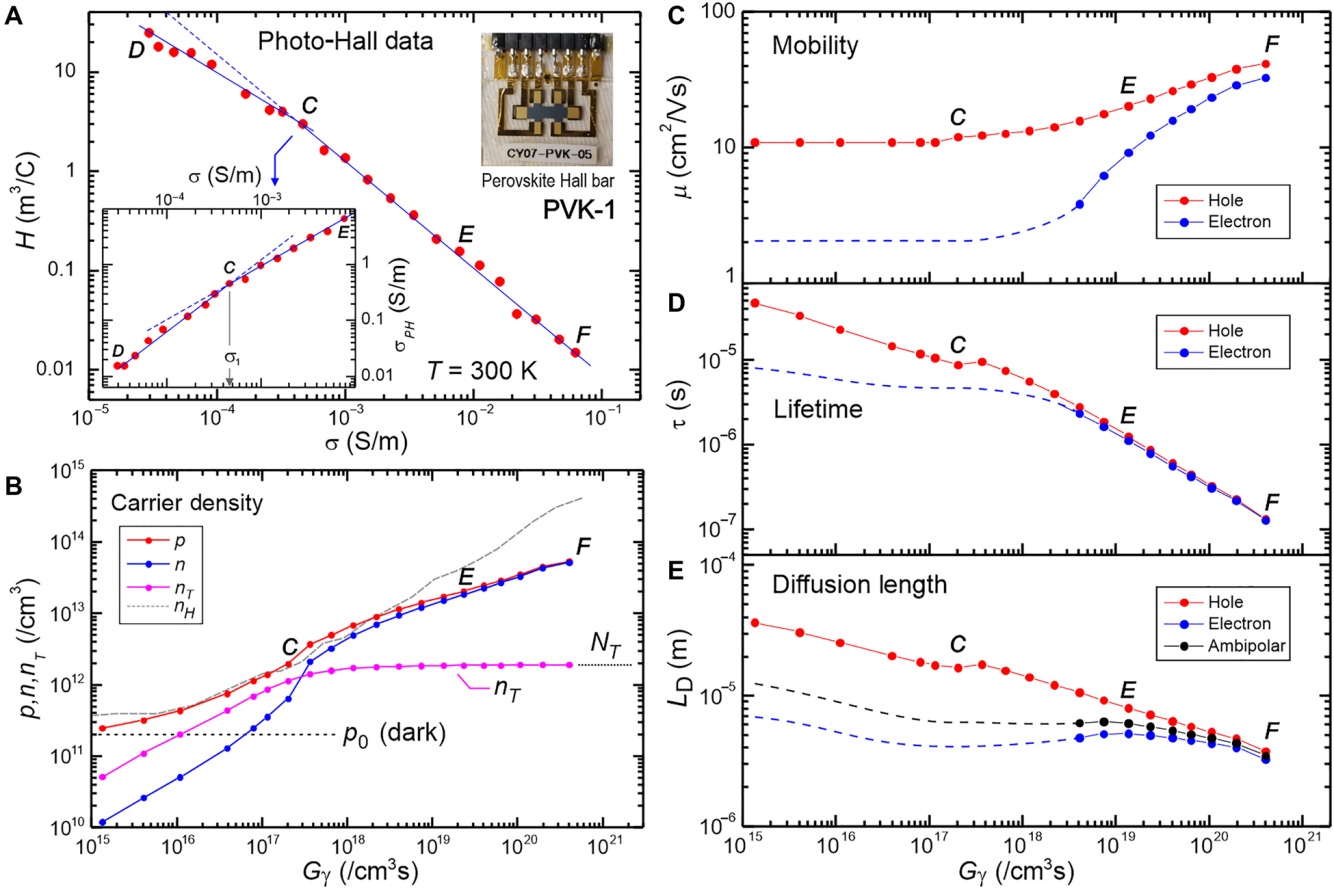
CTRPH trap analysis of a high-performance FAPbI_3_ perovskite film “PVK-1.” (**A**) The photo-Hall data with laser light λ = 615 nm and maximum intensity *I_L_* = 16 mW/cm^2^. Points D, C, and F mark the dark, inflection point, and full light intensity, respectively. Bottom inset: $\sigma_{\mathrm{PH}}$versus σ data that show bending behavior due to trap. Top inset: the perovskite Hall bar sample. (**B**) Carrier density versus absorbed photo density (*G*_γ_). $n_{H}=1/\mathrm{eH}$ is the Hall density for comparison. (**C**) Mobility. (**D**) Recombination lifetime. (**E**) Diffusion length. Dashed curves in (C) to (E) are estimated minority carrier values as the minority mobility values between dark and inflection point C are more uncertain.

We further apply the CTRPH technique to a high-performance FAPbI_3_ perovskite photovoltaic film, processed identically to the perovskite layers used in solar cell devices that recently achieved a record power conversion efficiency of 25.4% (*28*). The sample is a six-terminal Hall bar on glass with thickness *d* = 0.7 μm. The sample demonstrates a CTRPH trap analysis in a unique context where (i) $\mu_{P}>\mu_{N}$ (Fig. 1D, quadrant II), (ii) a high injection regime ($\Delta p\gg p_{0}$), and (iii) variable mobility at high light intensity. To address these characteristics, the analysis is divided into two regimes: trapping regime (indicated by data point range D-C-E in Fig. 3A) and high light intensity where the mobility may vary (range *E* to *F*). We observe that the perovskite data exhibit broad hyperbola or quadrant II behavior as shown in Fig. 1D where $\mu_{P}>\mu_{N}$, i.e., the minority (electron) mobility is less than that of majority (hole). The hyperbola appears broad because, at high light intensity (in the second segment), the minority carrier begins to contribute to the transport, but its contribution is small and only slightly changes the slope.

From the inflection point *C* in Fig. 3A (inset), we determine the trap density: *N*_*T*_ = 3.7 × 10^12^/cm^3^ using Eq. 3, which is ~30× smaller than Δ*n* or Δ*p* at maximum light intensity (see Fig. 3B). Notably, this *N*_*T*_ value is significantly below the detection limit of junction capacitance–based trap extraction methods (*N*_*T*,min_ ~10^15^/cm^3^ at *d* = 0.7 μm) (*7*, *29*), underscoring the advantage of this technique. This measurement also shows that this perovskite film has trap density on par with single crystal type (*7*) consistent with its high photovoltaics performance. The hyperbola curve profile is however too smooth to confidently determine the gap Δ and the trap energy level, but we estimate *E_TC_* ~ 0.3 to 0.7 eV.

In the high light intensity regime, we obtain self-consistent solutions using an iterative technique for intensity-dependent mobility, as described in our previous work (*21*) (see also section SD.4 for more detailed analysis). The extracted parameters are displayed as a function of absorbed photon density $G_{\gamma }$ in Fig. 3 (B to E). We observe that this perovskite sample exhibits excellent charge carrier parameters (μ ~ 40 cm^2^/Vs, τ ~ 100 ns and *L*_*D*_ ~ 3 μm) at maximum light intensity of ~0.16 sun, along with a very low trap density *N*_*T*_. This is consistent with the recent record performance of the associated solar cell device (*28*).

We note another aspect of this work: Our technique uses a simple model that assumes a single dominant trap with a narrow energy level. In practice, multiple traps or more complex situations—such as distributed traps, tail states, or Urbach tail—may exist, accompanied by a distribution of carrier mobilities. Nevertheless, we expect a qualitatively similar behavior to the single-level model, in which the $\sigma_{\mathrm{PH}}$ versus σ plot still consists of two segments corresponding to the trap-filling and trap saturated regime, which may not be exactly a hyperbola. For example, in the case of multilevel traps, we expect that the behavior of the corresponding $\sigma_{\mathrm{PH}}$ versus σ trace at a given temperature will be dominated by the deepest trap level which is partially filled. Therefore, if the trap energy levels are well separated, and we observe a hyperbola-like plot, we could use the single-level trap extraction formulas to obtain features of a multilevel trap distribution. These cases will be investigated in more detail in the future.

In summary, we have demonstrated a comprehensive approach for extracting trap and charge carrier properties from semiconductors using the CTRPH technique, which relies on a detailed analysis of the $\sigma_{\mathrm{PH}}$ versus σ curve, whose underlying behavior follows a newly derived hyperbola equation. This work provides a unified framework for gaining deeper insights into charge carrier transport and trapping effects by using the most common excitations: electric field, magnetic field, photon (light), and phonon (via lattice temperature control) based on the Hall effect. Beyond traditional electric and magnetic field applications in Hall measurements, increasing photon intensity allows us to fill traps, determine their density, and evaluate the mobility ratio of the charge carriers. By varying temperature or phonon excitation, we probe the trap energy levels. This technique significantly enhances the capabilities of Hall effect measurements and holds potential for broad application, enabling single-experiment, comprehensive evaluation of charge carrier transport and trap properties in many emerging advanced electronic materials.

## MATERIALS AND METHODS

### Experimental setup

The setup is shown in Fig. 1A. The PDL Hall system consists of a dry cryogen–free displex cryostat system with a custom-built cold stage equipped with a 50-W resistive heater, enabling temperature control from 20 to 340 K using a Lakeshore 340 temperature controller. The fundamental operation of the PDL Hall system is detailed in (*21*–*23*). The sample is mounted on the cold stage, positioned between two rotating PDL magnets made of NdFeB, each measuring 25.4 mm in diameter and 25.4 mm in length. The magnetic field applied to the sample is *B =* ±0.5 T.

Light illumination is provided by either a red-green-blue (RGB) laser or supercontinuum white laser (Super K Fianium FIU-15 from NKT Photonics) equipped with monochromator system. Light intensity is controlled over eight orders of magnitude using a combination of a continuous neutral density filter (CNDF) and optical density 4 (OD4) filter. The CNDF is driven by a stepper motor and the OD4 filter is driven by a two-position motorized flipper. A series of biconvex and biconcave lenses expand the beam to obtain ~10-mm coverage diameter to ensure uniform illumination of the entire Hall sample. The light beam is then split by a beam splitter, with one part directed downward to a “monitor photodetector” for real-time intensity monitoring and the other is directed at the sample.

The electronic instrumentation system consists of a Keithley 2450 source meter unit for providing current or voltage to the sample, a Keithley 2182A nanovoltmeter for measuring longitudinal and transverse voltage across the Hall sample, and a Keithley 6485 picoammeter to measure photocurrent or light intensity via the monitor photodetector or the reference photodetector cell. We have also built a custom PDL electronic control box that contains Raspberry Pi (RPi) Compute Module 4 microcomputer, a four-channel stepper motor controller (EZ4AXIS23WV from AllMotion), Pi-Plate data acquisition boards with analog and digital I/O mounted on top of the RPi, and custom-designed Hall effect switch matrix unit. This high-input impedance switch matrix (capable of resistance measurements up to 1 TΩ) features six sample input channels, two source input channels, and two voltage read-out channels, allowing simultaneous measurements of longitudinal (*R*_xx_) and/or transverse (*R*_xy_) resistance.

The stepper motor controller controls the rotation of the PDL magnets, the CNDF, and a linear stage for switching between the RGB laser and the Super K laser. The RPi microcomputer functions as a server, controlling operations of various modules inside the box. The box is controlled by a client Windows computer that runs MATLAB programs to execute various measurement operations.

### Fabrication of SOI Hall samples

The sample is an intrinsic SOI wafer, consisting of a 5-μm single-crystalline intrinsic Si device layer, a 500-nm SiO_2_ layer, and a 525-μm Si handle layer. To transfer this single-crystalline Si film onto a SiO_2_ substrate, the SOI wafer was bonded to a 500-μm-thick borosilicate glass using a fusion bonding process at 673 K and a pressure of 1.5 kN. Subsequently, the unwanted handle layer was removed via chemical mechanical polishing process, and residual Si was etched using a SF_6_ dry etch process. The remaining 500 nm SiO_2_ layer was then chemically etched with HF solution, yielding the single-crystalline Si film on the SiO_2_ substrate wafer. This Si/SiO_2_ wafer was then diced into 10 mm–by–10 mm pieces, and a photolithography process was used to form a Hall bar photoresist pattern on the Si film. The Si Hall bar was then created using SF_6_ dry etching, followed by photoresist removal. Last, 200-nm-thick Au electrodes were deposited on the Hall bar using an e-beam evaporator. The SOI Hall bar sample was mounted on a custom-made sample printed circuit board (PCB) package using “GE” varnish, and the Hall bar contacts were wire-bonded to the PCB pads. For variable temperature photo-Hall measurements, a small diode temperature sensor was attached to the glass substrate to accurately monitor the sample’s temperature.

### Fabrication of perovskite Hall samples

The perovskite Hall samples are based on formamidinium-rich lead iodide (FAPbI_3_) films with methylammonium chloride (MACl) additives. This high-quality perovskite exhibits a solar cell device power conversion efficiency of 25.7% and has a bandgap of 1.55 eV as reported in (*28*, *30*). Following these reports, FAPbI_3_ black powder was synthesized by first dissolving PbI_2_ and FAI in a 1:1 molar ratio in 2-methoxyethanol (0.8 M), and then filtered using a polyvinylidene fluoride filter with 0.45-μm pore size. The filtered solution was placed in a flask incubated in an oil bath at 120°C for 1 hour with slow stirring. The resulting black powder was filtered using a glass filter and dried at 60°C for 24 hours.

The perovskite precursor solution was prepared by dissolving 1550 mg FAPbI_3_ and 61 mg MACl with 1 ml of *N*,*N*′-dimethylformamide/dimethyl sulfoxide (4:1). The perovskite solution was filtered with a polyvinylidene fluoride filter (0.2 μm), and then 70 μl of the filtered perovskite solution was spread onto the 2.5 cm–by–2.5 cm glass substrate at 8000 rpm rotation speed for 50 s. During spin-coating, 1 ml of diethyl ether was dropped on the perovskite film at 10 s using a homemade pipette. The resulting film was annealed at 150°C for 15 min, followed by 100°C for 30 min on a hotplate, yielding high-quality FAPbI_3_ film with a thickness of 700 nm.

To perform photo-Hall measurement, a six-terminal perovskite Hall bar device was patterned by scraping away the excess film outside the Hall bar region. A 100-nm-thick Au contact pattern was then deposited on the film. An eight-terminal header pin was attached to the glass substrate with epoxy, and its metal pins were connected to the Au contact pattern using silver epoxy. A small diode temperature sensor was also attached to the sample to monitor its temperature. Since the perovskite thin film is highly sensitive to air and moisture, the Hall bar was encapsulated by attaching a secondary glass cover with epoxy. We attempted the measurements immediately after sample fabrication within a month and stored the samples in nitrogen desiccator box.

## Supplementary Materials

### This PDF file includes:

Supplementary Text Figs. S1 to S9 Tables S1 to S5 References

Correction (28 January 2026):
The original version of the Supplementary Materials PDF contained unintentional boldface font display in some equations and errors in the subpanel labels in the fig. S6 caption. The Supplementary Materials PDF has been corrected to resolve these issues. The original version is available here:
