# Supplementary material for: Electronic trap detection with carrier-resolved photo-Hall effect
Source: Sci Adv. 2026 Jan 1;12(1):eadz0460. doi: 10.1126/sciadv.adz0460 (PMC12757034; doi:10.1126/sciadv.adz0460)
Supplement: Supplementary file 2 — Correction (28 January 2026): The original version of the Supplementary Materials PDF contained unintentional boldface font display in some equations and errors in the subpanel labels in the fig. S6 caption. The Supplementary Materials PDF has been corrected to resolve these issues. The original version is available here: [file sciadv.adz0460_sm.v1.pdf]

Science Advances
AAAS

Supplementary Materials for

Electronic trap detection with carrier-resolved photo-Hall effect

Oki Gunawan *et al.*

Corresponding author: Oki Gunawan, ogunawa@us.ibm.com; Byungha Shin, byungha@kaist.ac.kr

Sci. Adv. **12**, eadz0460 (2026)
DOI: 10.1126/sciadv.adz0460

This PDF file includes:

Supplementary Text
Figs. S1 to S9
Tables S1 to S5
References

A. Theory of Carrier and Trap Resolved Photo-Hall Effect

A.1 Derivation

We will derive a general relationship of photo-Hall effect in the presence of trap that capture the role of four excitations — electric field, magnetic field, photon and phonon — in a single equation, and in the process, allowing us to extract almost all charge carrier and trap parameters in the material (see section C). The electric field (or electric current) and magnetic field excitations have been used in the traditional Hall effect which allows us to obtain the electrical conductivity σ and Hall coefficient H. The key theoretical innovation in this work is the introduction of a new quantity called photo Hall conductivity (PHC) that will significantly simplify many calculations. The PHC is defined as:

$$\sigma_{PH} = \frac{\sigma^2 H}{r\mu_0}, \tag{S1}$$

where r is the Hall scattering factor which can be assumed to be equal to 1 in most cases (*24*), and μ_0 is the majority carrier mobility. This concept is rooted in our earlier finding (*21*) that the hole-electron mobility difference can be obtained from the slope of $\sigma^2 H$ vs. σ plot, i.e.: $\Delta\mu_H = d(\sigma^2 H)/d\sigma$, which assumes negligible trapping effect. In the presence of increasing photon intensity, σ generally increases and σ_{PH} will also change depending on the trapping effect and the carrier mobilities. By changing lattice temperature or phonon excitation we can alter the σ_{PH} vs. σ behavior that allows us to probe the trap energy level. We assume that electron and hole mobilities are constant throughout the process.

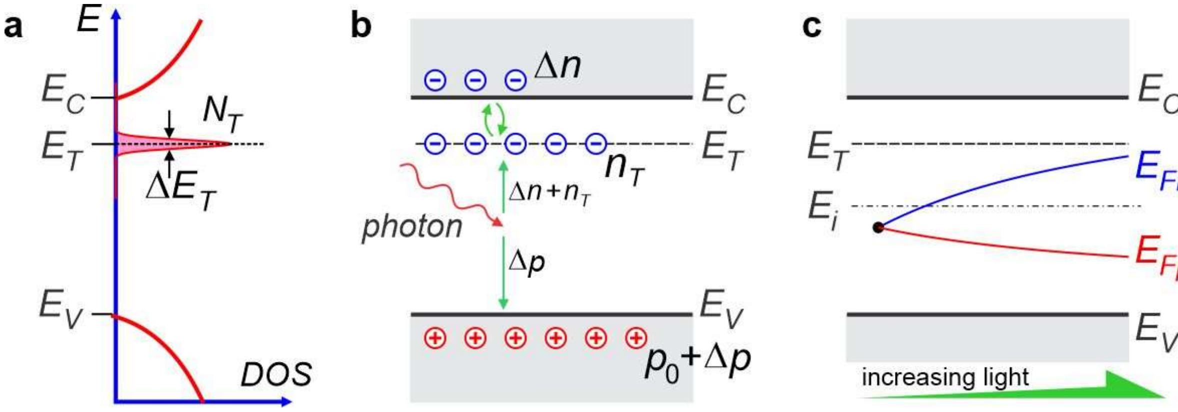

Figure S1. A model of a single level trap in a *P*-type semiconductor with light: (a) Density of states energy diagram. **(b)** The energy band diagram of a semiconductor and a single-level trap with photon excitation. **(c)** Quasi-Fermi levels for electron (blue) and hole (red) with increasing light intensity. E_T and E_i are the trap and intrinsic energy level respectively.

We start with single trap Shockley-Read-Hall model (*31, 32*) for a semiconductor that describes the process of recombination and generation of charge carriers (electrons and holes) in a

semiconductor via trap states (*24*) as shown in Fig. S1. The trap states arise from imperfections in the semiconductor crystal structure, impurities or defects. To illustrate the carrier and trap-resolved photo Hall effect calculation we a use a model based on P-type semiconductor, a similar model for N-type material is provided in section A.5. We start with a single level trap close to the conduction band (CB), thus the trap is more sensitive to electron (the minority carrier). We recognize that, in reality, there could be more than one trap present, and they could be located anywhere in the bandgap, however it is sufficient to model the most dominant trap that has the most impact to the charge-transport process, i.e., the minority carrier trap whose energy level is deep but closer to conduction band rather than the valence band (VB).

The trap has a density of state (DOS) $g_D(E)$ described by a Dirac-delta function centered at energy E_T and with a total trap density N_T:

$$g_D(E) = N_T \delta(E - E_T)\,. \tag{S2}$$

With increasing light intensity, the Fermi level splits into electron and hole quasi-Fermi levels as shown in Fig. S1c. Given the electron quasi-Fermi level E_{Fn} at certain light intensity we can calculate the density of the electron in the trap n_T as:

$$n_T = \int_0^\infty g_D(E) f_{FD}(E)\, dE = \frac{N_T}{1 + g_T \exp\left((E_T - E_{Fn})/k_B T\right)}\,, \tag{S3}$$

where $f_{FD}(E) = 1/[1 + g_T \exp((E - E_F)/k_B T)]$ is the Fermi-Dirac distribution function that includes the trap level degeneracy factor g_T, E_F is the Fermi energy level, k_B is the Boltzmann constant, and T is the temperature. The trap degeneracy factor g_T depends on the charge state and the nature of the traps and is generally difficult to determine, however it can be assumed to be one for most deep level traps (*26*). The impact of g_T can be viewed as introducing an offset in the E_T determined from the experiment, e.g. Eq. (S3) can also be written as: $n_T = N_T/\left(1 + \exp\left[\left(E_T^{'} - E_{Fn}\right)/k_B T\right]\right)$ where $E_T^{'} = E_T - k_B T \ln g_T$. However, the offset is small, e.g. for $g_T = 2$ at $T = 300$ K we have $k_B T \ln g_T = 18$ meV.

We further assume non-degenerate case where E_{Fn} is sufficiently far away from CB ($E_C - E_{Fn} \gg k_B T$) such that Boltzmann statistic can be applied. This is a good approximation, e.g. in Si for a maximum case of $\Delta n \sim 10^{15}$ /cm^3 under 1 sun illumination, we have $E_C - E_{Fn} {\sim} 10\, k_B T$. We calculate Δn using:

$$\Delta n = N_C \exp[-(E_C - E_{Fn})/k_B T]\,, \tag{S4}$$

where N_C is the effective DOS of the CB (*24*):

$$N_C = 2\left(\frac{m_{d,e}^{*} k_B T}{2\pi\hbar^2}\right)^{3/2}\,, \tag{S5}$$

with $m_{d,e}$* is the DOS effective mass of electron and $\hbar$ is the reduced Planck constant. By eliminating E_{Fn} from Eq. (S3) and (S4), we obtain a nonlinear relationship between n_T and Δn:

$$n_T \Delta n + g_T N_C n_T e^{-E_{TC}/k_B T} - N_T \Delta n = 0\,, \tag{S6}$$

where $E_{TC} = E_C - E_T$ is the depth of trap energy level from the CB edge (a positive number).

Next, we evaluate the conductivity and Hall coefficient in a bipolar transport process (*1, 33*) at relatively low magnetic field: $B < 1/\mu$, where B is the magnetic field and μ is the charge carrier mobility:

$$\sigma = e(\mu_N n + \mu_P p) \tag{S7}$$

$$H = \frac{r(p-\beta^2 n)}{(p+\beta n)^2 e}, \tag{S8}$$

where $\beta = \mu_N / \mu_P$ is the electron to hole mobility ratio, p is the free hole density: $p = p_0 + \Delta p$ and n is the free electron density: $n = n_0 + \Delta n \simeq \Delta n$, since $n_0 = n_i^2 / p_0 << \Delta n$. We have Δn, Δp, n_0 and p_0 as the photocarrier and equlibrium (dark) carrier density for electron and hole respectively.

The charge neutrality stipulates that the photogeneration process yield equal amount of holes and electrons, with some of the electrons go to the trap (*34*):

$$\Delta p = \Delta n + n_T - n_{T,0} \tag{S9}$$

where $n_{T,0}$ is the equilibrum (dark) electron density in the trap, given as:

$$n_{T,0} = N_T / (1 + g_T \exp[-(E_T - E_{Fp}) / k_B T]) \tag{S10}$$

Now we can express p as:

$$p = p_0 + \Delta p = p_0 - n_{T,0} + \Delta n + n_T \simeq p_0 + \Delta n + n_T . \tag{S11}$$

Here we can ignore $n_{T,0}$ by assuming $p_0 \gg n_{T,0}$, which is generally true if the trap level (E_T) is away from the hole Fermi energy (E_{Fp}) by few $k_B T$'s. If this condition is not satisfied, i.e., when E_T is close to E_{Fp}, we can replace p_0 in s_N parameter [Eq. (S17)] with $p_0 - n_{T,0}$, where $n_{T,0}$ is calculated using Eq. (S10). Iteration to achieve self consistent solutions for N_T and E_T may be necessary.

Now Eq. (S7) and (S8) can be expressed as:

$$\sigma = \sigma_0 + e\mu_P (n_T + (1+\beta)\Delta n) \tag{S12}$$

$$\sigma_{PH} = \sigma_0 + e\mu_P (n_T + (1-\beta^2)\Delta n), \tag{S13}$$

where: $\sigma_0 = e\mu_0 p_0$ is the dark conductivity in the dark and μ_0 is the mobility of the majority carrier in the dark. This is a linear system of two equations with two unknowns: n_T and Δn, with solutions:

$$n_T = \frac{(\beta-1)\sigma + \sigma_{PH} - \beta\sigma_0}{e\mu_P \beta} \tag{S14}$$

$$\Delta n = \frac{\sigma - \sigma_{PH}}{e\mu_P \beta(\beta+1)} . \tag{S15}$$

Substituting these expressions to Eq. (S6) we obtain:

$$(\beta-1)\sigma^2 + (2-\beta)\sigma\sigma_{PH} - \sigma_{PH}^2 + \beta[(\beta-1)s_E - s_N]\sigma + \beta(s_N + s_E)\sigma_{PH} - \beta^2\sigma_0 s_E = 0 , \tag{S16}$$

here we define the parameters:

$$s_N = e\mu_0(p_0 + N_T) \quad \text{and} \quad s_E = e\, g_T \mu_0 (\beta+1) N_C \exp(-\varepsilon_{TC}) , \tag{S17}$$

with $\epsilon_{TC} = (E_C - E_T)/k_B T$ is the trap energy below CB edge normalized by the thermal energy $k_B T$. As mentioned in the main text, the parameter s_N and s_E have very clear physical and geometrical meaning: s_N depends on N_T and determines the horizontal peak (or vertex) position of the hyperbola, while s_E depends on E_T and determines the vertical peak position (see also Fig. S3).

We note that Eq. (S16) follows a generalized conic section equation (*35*):

$$Q(x,y) = Ax^2 + Bxy + Cy^2 + Dx + Ey + F = 0 , \tag{S18}$$

where (x, y) are (σ, σ_{PH}) and:

$$\begin{aligned} &A = \beta - 1, && B = 2 - \beta, && C = -1 \\ &D = \beta[(\beta-1)s_E - s_N], && E = \beta(s_N + s_E), && F = -\beta^2 \sigma_0 s_E \end{aligned} \tag{S19}$$

We will show in the next section that Eq. (S16) is guaranteed to be a hyperbola. Next, we can solve the quadratic equations in Eq. (S16) and (S18) to obtain σ_{PH} as a function of σ:

$$y(x) = \left[-Bx - E \pm \sqrt{(Bx+E)^2 - 4C(Ax^2 + Dx + F)} \right] / 2C \tag{S20}$$

$$\sigma_{PH}(\sigma) = \left[\beta(s_N + s_E) + (2-\beta)\sigma - \beta\sqrt{(s_N + s_E + \sigma)^2 - 4(s_E\sigma_0 + s_N\sigma)} \right] / 2 , \tag{S21}$$

which we present as Eq. 2 in the main text. The positive branch of the quadratic solution is discarded as it corresponds to negative conduction band densities Δn.

It is useful to find the two asymptotic lines of the hyperbola as $\sigma \to \pm\infty$ from Eq. (S21). They are:

$$\sigma_{PH}\big|_{\sigma\to-\infty} = \beta s_E + \sigma \tag{S22}$$

$$\sigma_{PH}\big|_{\sigma\to\infty} = \beta s_N + (1-\beta)\sigma . \tag{S23}$$

We note an important feature of this hyperbola equation, the slope $d\sigma_{PH}/d\sigma$ of the two asymptotes are 1 and $1-\beta$ respectively. They are indicated in Fig. 1d (quadrant I and II) for the case of P-type materials. There is a related observation, if we calculate the slope: $s = d(\sigma^2 H)/d\sigma = r\mu_0 (d\sigma_{PH})/d\sigma$, we will obtain $s = \mu_0$ and $s = \Delta\mu = \mu_P - \mu_N$ for the "*trap-filling*" and "*trap-full*" regime respectively. This means that in the first (second) regime the slope yields the majority

carrier mobility (the mobility difference). This is what we observe in Fig. 1b and discussed in the main text.

We also have an interesting case in the low temperature limit where ϵ_{TC} is large or $s_E \to 0$. Here Eq. (S21) becomes a degenerate hyperbola that appears as two lines, which is given as:

$$\sigma_{PH}(\sigma) = \left[\beta s_N + (2-\beta)\sigma - \beta\sqrt{(\sigma - s_N)^2}\right]/2 = \left[\beta s_N + (2-\beta)\sigma - \beta\,|\,\sigma - s_N\,|\right]/2\,, \qquad \text{(S24)}$$

which can also be written as:

$$\sigma_{PH}(\sigma) = \begin{cases} \sigma & \sigma \le s_{\mathrm{N}} \\ \beta\, s_N + (1-\beta)\sigma & \sigma > s_{\mathrm{N}} \end{cases} \qquad \text{(S25)}$$

The plot of this function is shown as lines DCF in Fig. S3b. This plot exhibits a peak or center point at $C\ (s_N, s_N)$.

A.2 The Photo-Hall Hyperbola Equation

It is very useful to simplify the general photo-Hall equation with trap in Eq. (S16), to a canonical hyperbola equation:

$$\frac{\sigma'^{2}_{PH}}{a^2} - \frac{\sigma'^{2}}{b^2} = 1\,. \qquad \text{(S26)}$$

Here we use the coordinate $(\boldsymbol{x}, \boldsymbol{y})$ and its rotated coordinate $(\boldsymbol{x}', \boldsymbol{y}')$ to represent $(\boldsymbol{\sigma}, \boldsymbol{\sigma_{PH}})$ and $(\boldsymbol{\sigma}', \boldsymbol{\sigma_{PH}}')$ respectively. The *x*'-*y*' axes represent the principal axes of the hyperbola. This is a hyperbola equation with vertical transverse axis (which opens toward up and down direction). The coordinate $(\boldsymbol{x}', \boldsymbol{y}')$ can be calculated from the translation and rotation of (*x*, *y*).

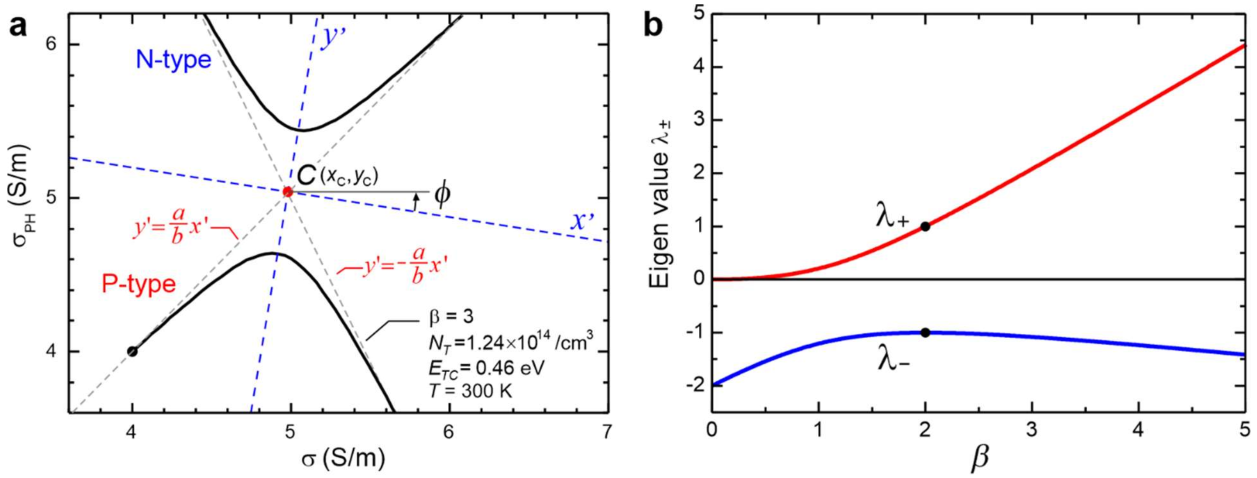

Figure S2. The photo-Hall Hyperbola plot and its matrix eigenvalues. (a) The hyperbola plot and the rotated (*x*'-*y*') coordinate frame. The lower (upper) hyperbola corresponds to a *P*-type (*N*-

type) system. **(b)** Eigen values of the main hyperbola quadratic matrix as a function of β. The black dots mark the special case for $\beta = 2$ where the eigen values are: $\lambda_\pm = \pm 1$ and $\phi = 0$.

We start with the basic conic section or quadratic equation [Eq. (S16) and (S18)] and express it in the matrix form as (*35*):

$$\mathbf{x}^T A_Q \mathbf{x} = 0, \tag{S27}$$

where, A_Q is called the quadratic equation matrix:

$$A_Q = \begin{bmatrix} A & B/2 & D/2 \\ B/2 & C & E/2 \\ D/2 & E/2 & F \end{bmatrix} \text{ and } \mathbf{x} = \begin{bmatrix} x \\ y \\ 1 \end{bmatrix}. \tag{S28}$$

Of special importance is the sub-matrix A_{33}, which is the characteristic matrix of the conic section. Its determinant is called the *discriminant* of the conic section (*36*), which will determine the type of conic section, i.e., whether it is an ellipse, parabola or hyperbola:

$$A_{33} = \begin{bmatrix} A & B/2 \\ B/2 & C \end{bmatrix}. \tag{S29}$$

For our photo-Hall problem, we have:

$$|A_{33}| = AC - B^2/4 = -\beta^2/4. \tag{S30}$$

Thus, it is always negative, and our conic section is guaranteed to be a hyperbola (*35*). This matrix also has eigenvalues that are important parameters in this model:

$$\begin{vmatrix} A-\lambda & B/2 \\ B/2 & C-\lambda \end{vmatrix} = 0, \tag{S31}$$

which yields:

$$\lambda_\pm = \left(C + A \pm \sqrt{(A-C)^2 + B^2}\right)/2 = (\beta - 2 \pm \sqrt{2\beta^2 - 4\beta + 4})/2. \tag{S32}$$

There are two eigenvalues, and they only depend on β. The plot is given in Fig. S2b.

The coordinate (x', y') can be calculated by applying a translation to point $C(x_C, y_C)$, followed by a rotation of $-\phi$ to the original coordinate system (x, y):

$$\begin{bmatrix} x' \\ y' \end{bmatrix} = \begin{bmatrix} \cos\phi & \sin\phi \\ -\sin\phi & \cos\phi \end{bmatrix} \begin{bmatrix} x - x_C \\ y - y_C \end{bmatrix}. \tag{S33}$$

Point C (x_C, y_C) is the center point of the hyperbola which can be calculated from the condition that it is a point where the gradient of the quadratic function Q in Eq. (S18) vanishes (*37*, *38*). A

way to view this problem is to consider a function $z = Q(x, y)$. If it is an ellipse, the center point would be the maximum or minimum of z. If it is a hyperbola, the center point would be the saddle point. In both cases, the center point is where $\nabla Q(x, y) = [0,0]$, therefore:

$$\nabla Q(x_C, y_C) = \begin{bmatrix} \partial Q / \partial x \\ \partial Q / \partial y \end{bmatrix} = \begin{bmatrix} 0 \\ 0 \end{bmatrix} \quad \text{or} \quad \begin{bmatrix} 2A\, x_C + B\, y_C + D \\ B\, x_C + 2C y_C + E \end{bmatrix} = \begin{bmatrix} 0 \\ 0 \end{bmatrix}. \tag{S34}$$

Now, we can solve for x_C and y_C:

$$\begin{bmatrix} x_C \\ y_C \end{bmatrix} = \frac{1}{4AC - B^2} \begin{bmatrix} BE - 2CD \\ BD - 2AE \end{bmatrix} = \begin{bmatrix} s_N - s_E \\ s_N + (\beta - 1) s_E \end{bmatrix}. \tag{S35}$$

This solution which defines the coordinate of center point *C*, plays a very important role in this model as it allows us to determine trap density N_T and trap energy level E_T.

The semi major and semi minor axis *a* and *b* are given as (*35*):

$$a^2 = -\frac{|A_Q|}{\lambda_+ \lambda_-^{\,2}} \quad \text{and} \quad b^2 = -\frac{|A_Q|}{\lambda_+^{\,2} \lambda_-}, \tag{S36}$$

where $|A_Q|$ is the determinant of A_Q: $|A_Q| = (4ACF - AE^2 - B^2F + BDE - CD^2)/4$. We define:

$$K = \frac{|A_Q|}{\lambda_+ \lambda_-} = \frac{CD^2 - BDE + AE^2}{B^2 - 4AC} + F = \beta^2 s_E (s_N - \sigma_0) = e\mu_0 \beta^2 N_T s_E \,. \tag{S37}$$

Note that the value *K* is always positive. Now we have:

$$a = \sqrt{-K/\lambda_-} \qquad \text{and} \qquad b = \sqrt{K/\lambda_+}\,. \tag{S38}$$

Next, we calculate the rotation angle ϕ of the hyperbola's principal axes as shown in Fig. S2a. We can derive this angle in a simple way by noting that the principal axes of the hyperbola bisects the asymptotes. We can determine the asymptotes of the hyperbola as $x, y \to \pm\infty$, thus only the first three terms dominate in the hyperbola equation in Eq. (S18):

$$A\, x^2 + B\, x\, y + C\, y^2 = 0\,. \tag{S39}$$

We can obtain the asymptote line equations by solving for *y*:

$$y = \frac{-B \pm \sqrt{B^2 - 4AC}}{2C} x\,, \tag{S40}$$

which implies that the slope of the asymptotes are: $m_{1,2} = (-B \pm \sqrt{B^2 - 4AC})/2C$. From Fig. S2a, we see that the principal axes form an angle ϕ with respect to the *x*-axis which bisects the two

asymptotes. In other words, ϕ is the average of the angles of the two asymptotes: ϕ_1 and ϕ_2. we have:

$$2\phi = \phi_1 + \phi_2 , \tag{S41}$$

where $\tan \phi_{1,2} = m_{1,2}$. Therefore, we have:

$$\tan 2\phi = \tan(\phi_1 + \phi_2) = \frac{\tan\phi_1 \tan\phi_2}{1 - \tan\phi_1 \tan\phi_2} = \frac{m_1 + m_2}{1 - m_1 m_2} = \frac{B}{A-C} = \frac{2-\beta}{\beta}, \text{ or:} \tag{S42}$$

$$\phi = \tfrac{1}{2}\tan^{-1}(2/\beta - 1) \tag{S43}$$

We also note another important characteristic, the ratio of b and a determines the curvature or the eccentricity (ϵ) of the hyperbola. The eccentricity of the hyperbola is given as (*36*):

$$\varepsilon = \sqrt{1 + (b/a)^2} , \tag{S44}$$

which depends on ratio of b and a, and in our CRPH problem, it solely depends on β:

$$\left(\frac{b}{a}\right)^2 = \sqrt{\frac{-\lambda_-}{\lambda_+}} = \frac{\beta - 2 - \sqrt{2\beta^2 - 4\beta + 4}}{\beta - 2 + \sqrt{2\beta^2 - 4\beta + 4}} . \tag{S45}$$

The eccentricity, ranging from 1 to ∞, determines the curvature of the hyperbola. The hyperbola is sharp when ε is low (~1) or β is large, and the hyperbola is broad when ε is large or β is very small ($\rightarrow 0$).

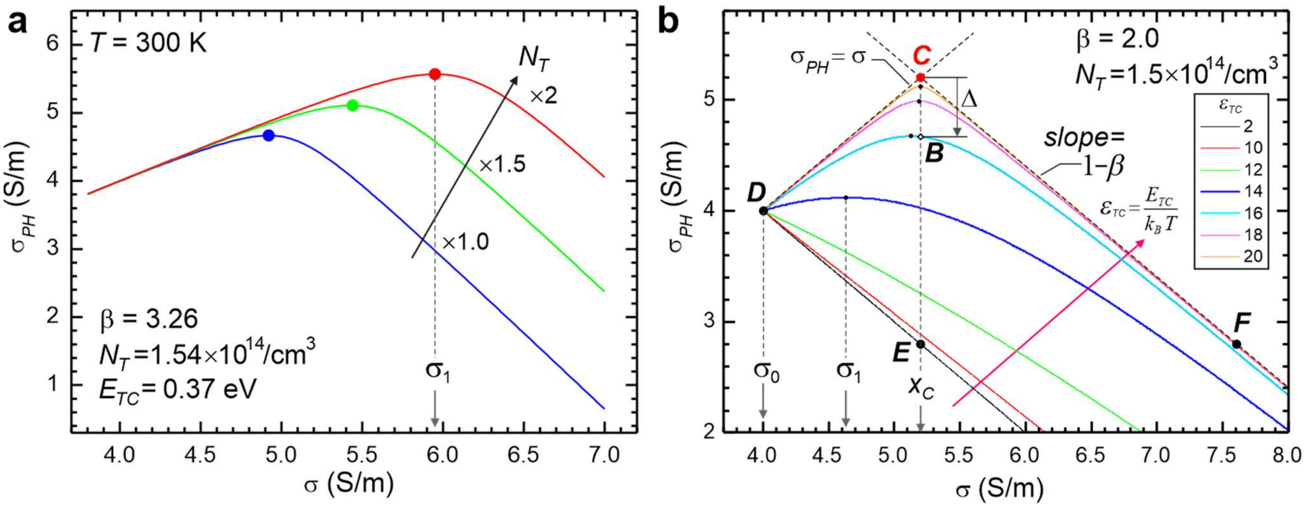

Figure S3. **Simulation of the impact of trap parameter N_T and E_T in a *P*-type material.** (a) Effect of increasing N_T that shifts the hyperbola peak to the right at fixed temperature. (b) Effect of increasing ε_T that makes the hyperbola sharper.

In summary, we have expressed the relationship between σ_{PH} vs. σ as a compact hyperbola equation in Eq. (S26), and elaborate its parameters such as x_C, y_C, ϕ, a and b in terms of the physical parameters β, N_T and E_T. In Fig. S3 we simulate a set of hyperbola curves where N_T and E_T vary. We make few notable remarks about the photo-Hall hyperbola characteristics:

1. Increasing N_T will shift the hyperbola peak (or vertex) to the right as shown in Fig. S3a, as it will take higher light intensity and photo carrier density to fill all the traps, after which the curve starts to turn around.

2. Increasing ϵ_{TC}, which occur when E_{TC} is larger or T is lower, will make the hyperbola sharper (see Fig. S3b) and approaching the two asymptotic lines. This is what we observe in Fig. 2 for the p-SOI data, where the $\sigma_{PH} - \sigma$ hyperbola curves get sharper at lower temperature.

3. The center point C plays a crucial role to determine N_T and E_T (see section A.3 and A.4). Point C is determined from the intersection of $\sigma_{PH} = \sigma$ line and the second asymptote line. This implies that we need to take enough data points at high light intensity to obtain the second asymptote. We note that the line: $\sigma_{PH} = \sigma$ is not necessarily the first asymptote at high temperature, but it becomes the first asymptote at low temperature.

4. If there is no trap, or the trapping effect is negligible, i.e., $N_T = 0$ or $E_T = 0$, the first segment of the hyperbola curve is absent, instead we only have the second segment with slope $1 - \beta$ (e.g. line DE in Fig. S3b).

5. We note a special case when $\beta = 2$. From Eq. (S43) we have $\phi = 0$, in other words, the principal axes of the hyperbola is already aligned with the x and y axes and thus no rotation is needed and (σ', σ_{PH}') is equal to (σ, σ_{PH}). This is also consistent with the fact that the slopes of the two asymptotes are: 1 and $1 - \beta = 1$ respectively, implying that the hyperbola is already symmetric in the original axes. We also have the eigen values: $\lambda_\pm = \pm 1$ and thus $a = b = K$. In this case, the $\sigma_{PH} - \sigma$ relationship is reduced to a very simple equation: $\sigma_{PH}^2 = \sigma^2 + K^2$.

6. The hyperbola bending is the sharpest when the minority carrier mobility is larger than that of the majority (i.e., quadrant I and IV in Fig. 1d). This is reasonable, because once the trap is full and the minority carrier start to contribute to the transport, the change in σ_{PH} is more significant if the minority carrier mobility is larger.

7. It is fascinating that the rich physics of the photo-Hall effect with trap can be captured by a succinct, hyperbola equation. Conic section solutions such as ellipse and hyperbola are well known in physics to describe motions of celestial objects. Recently, the hyperbolic trajectory of asteroid “1I/’Oumuamua”, characterized by its unprecedentedly high eccentricity (ε =1.2), played a decisive role in confirming its groundbreaking identification as the first known interstellar object to visit our solar system, as reported by Meech *et al.* in Nature (*39*).

A.3 Determination of Trap Density (N_T)

From the hyperbola model, we can determine the trap density in a simple way using the peak (or the vertex) coordinate. The *x*-coordinate of the peak is close to that of center point *C*: $x_C = s_N - s_E$. We note that, in the limit: $\epsilon \to \infty$, which occurs at low temperature, the hyperbola becomes very sharp and we have: $s_E \sim 0$ and x_C becomes close to the peak position: $x_C \simeq \sigma_1$. Using Eq. (S17) we have $\sigma_1 = s_N = \sigma_0 + e\mu_0 N_T$. Therefore we can solve for N_T and obtain the simple formula below. We denote $\tilde{N}_T$ as the estimated trap density.

$$\tilde{N}_T = \frac{\sigma_1 - \sigma_0}{e\mu_0}. \tag{S46}$$

Temperature-dependent effect in N_T determination:

Strictly speaking, the N_T determination from the hyperbola peak (or inflection point) using Eq. (S46) contains some temperature-dependent factor, which can be derived below. The peak position can be calculated from Eq. (S21):

$$\left.\frac{d\sigma_{PH}}{d\sigma}\right|_{\sigma=\sigma_1} = 1 - \frac{\beta}{2} - \frac{\beta(\sigma - s_N + s_E)}{2\sqrt{\sigma^2 - 2(s_N - s_E)\sigma + (s_N + s_E)^2 - 4\sigma_0 s_E}} = 0 \tag{S47}$$

$$\sigma_1^{\ 2} - 2(s_N - s_E)\sigma_1 + (s_N + s_E)^2 - 4\sigma_0 s_E + \frac{\beta^2}{1-\beta} e\mu_0 N_T s_E = 0. \tag{S48}$$

Solving this quadratic equation, we obtain, the peak position:

$$\sigma_1 = (s_N - s_E) - (\beta - 2)\sqrt{\frac{e\mu_0 N_T s_E}{\beta - 1}}. \tag{S49}$$

So now we have the exact expression for the $\tilde{N}_T$:

$$\tilde{N}_T = \frac{\sigma_1 - \sigma_0}{e\mu_0} = N_T - (1+\beta) g_T N_C \exp(-\varepsilon_{TC}) - |\beta - 2| \sqrt{\frac{\beta+1}{\beta-1} N_T N_C g_T \exp(-\varepsilon_{TC})}. \tag{S50}$$

We note that the effective density of states N_C contains temperature dependent factor $T^{3/2}$. We can write this equation as:

$$\tilde{N}_T(T) = N_T - a_1 u(T) - a_2\sqrt{u(T)}, \tag{S51}$$

where $u(T) = T^{3/2}\exp(-\epsilon_{TC})$ is the temperature dependent factor and constants: $a_1 = 2 g_T(\beta + 1)\ (m_{d,e}^{\ *} k_B / 2\pi\hbar^2)^{3/2}$ and $a_2 = |\beta - 2| \sqrt{g_T(\beta+1) N_T / (\beta - 1)} \times (m_{d,e}^{\ *} k_B / 2\pi\hbar^2)^{3/4}$. This equation predicts that $\tilde{N}_T = N_T$ at low temperature and as the temperature increases $\tilde{N}_T$ will drop. This effect is observed in Fig. 2b where $\tilde{N}_T$ drops at higher temperature, and the actual N_T can be determined from the lowest temperature data.

A.4 Determination of Trap Energy Level (E_T)

E_T determination from a single temperature measurement:

We can determine the trap energy level from the gap parameter "Δ". We note that Δ is very close to the semimajor axis a, in fact, if ϕ = 0, the gap is exactly the same as the hyperbola semi-major axis, i.e., $\Delta = a$. The gap Δ can be calculated as the distance between point C and the hyperbola curve at point $\sigma = x_C$:

$$\Delta = y_C - \sigma_{PH}(x_C) = \beta\sqrt{s_E(s_N - \sigma_0)} = \beta\sqrt{e\mu_0 N_T s_E}\,. \tag{S52}$$

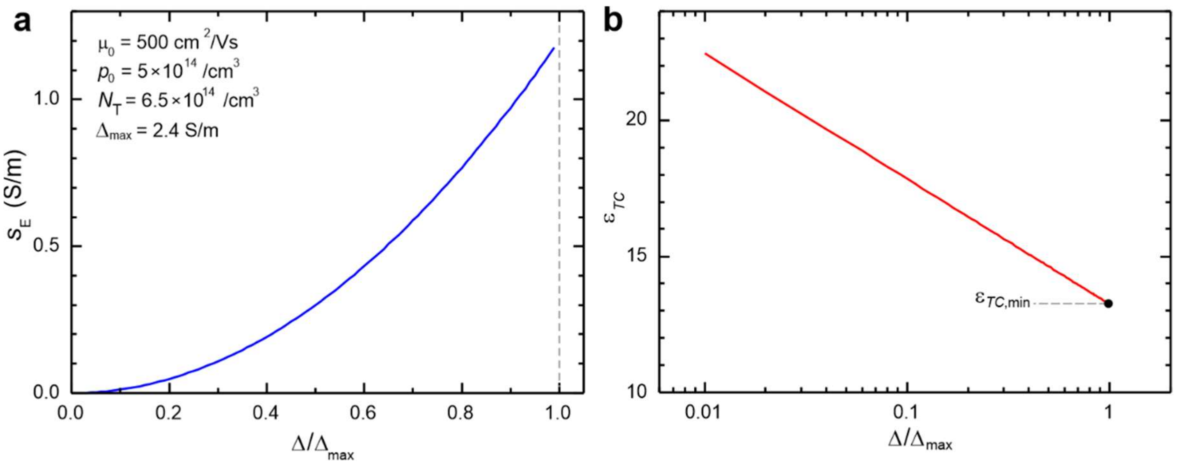

Figure S4. Determination of E_T from the gap Δ. (a) s_E vs. Δ plot. (b) Determination of $\boldsymbol{\epsilon_{TC}}$ from Δ.

The maximum gap Δ_{max} can be obtained from the segment CE in Fig. S3b. Point C is obtained when $\epsilon \to \infty$ or $s_E = 0$. Point E is from segment DE which occurs when $\epsilon \to 0$ or $s_E \gg s_N$. In this limit Eq. (S21) reduces to:

$$\tilde{\sigma}_{PH}(\sigma) = (1-\beta)\sigma + \beta\sigma_0\,, \tag{S53}$$

which is the equation for line DE. We obtain a simple expression:

$$\Delta_{max} = y_C - y_E = s_N - \tilde{\sigma}_{PH}(s_N) = e\beta\mu_0 N_T\,. \tag{S54}$$

We can calculate the parameter s_E as:

$$s_E = \frac{\Delta^2}{e\beta^2\mu_0 N_T}\,. \tag{S55}$$

From Eq. (S17), we can solve for E_T as a function of Δ :

$$E_{TC} = k_B T \ln\left(\frac{e\mu_0(1+\beta)N_C}{s_E} \right) = k_B T \ln\left(\frac{e^2 g_T \mu_0{}^2 \beta^2 (1+\beta) N_C N_T}{\Delta^2} \right). \quad \text{(S56)}$$

If we substitute $\Delta = \Delta_{max}$ we obtain the minimum trap energy level that can be detected as shown in Fig. S4b which is given as:

$$E_{TC,\min} = k_B T \ln\left[g_T (1+\beta) N_C / N_T \right]. \quad \text{(S57)}$$

This equation implies that: (1) there is a minimum energy level that we can detect at a given temperature, (2) we can detect smaller trap energy level at lower temperature. We can plot s_E vs. Δ/Δ_{max} from Eq. (S55) as shown in Fig. S4a, we see that s_E increases monotonically with Δ. The plot of trap energy vs. Δ is shown in Fig. S4b and the $\epsilon_{TC,min}$ is also indicated.

$\boldsymbol{E_T}$ determination from variable temperature measurements:

Besides using the gap "Δ", we can also utilize temperature-dependent slope of $S = \mathrm{d}\sigma_{PH}/\mathrm{d}\sigma$ in the dark to extract E_T. We start from a series expansion of $\sigma_{PH}(\sigma)$ in terms of $\sigma - \sigma_0$:

$$\sigma_{PH}(\sigma) = \sigma_0 + \left[1 - \frac{\beta s_E}{s_N + s_E - \sigma_0} \right] (\sigma - \sigma_0) + \mathrm{O}\left[(\sigma - \sigma_0)^2 \right]. \quad \text{(S58)}$$

We define the slope in the dark $S_0 = \mathrm{d}\sigma_{PH}/d\sigma|_{\sigma=\sigma_0}$ as:

$$S_0 = \left. \frac{d\sigma_{PH}}{d\sigma} \right|_{\sigma=\sigma_0} = 1 - \frac{g_T \beta(\beta+1) N_C \exp(-\varepsilon_{TC})}{N_T + g_T(\beta+1) N_C \exp(-\varepsilon_{TC})}. \quad \text{(S59)}$$

We can rearrange this expression and isolate the exponential factors, and express the effective DOS [Eq. (S5)] in terms of its temperature-dependent factor, i.e., $N_C = N_{C0} T^{1.5}$:

$$\frac{g_T N_0 T^{1.5}}{N_T} (\beta+1) \left[\frac{\beta}{1-S_0} - 1 \right] = \exp(\varepsilon_{TC}) \quad \text{(S60)}$$

$$\frac{1-S_0}{(\beta+1)(\beta-1+S_0) T^{1.5}} = \frac{g_T N_{C0}}{N_T} \exp(-\varepsilon_{TC}) \quad \text{(S61)}$$

$$\ln\left(\frac{1-S_0}{(\beta+S_0-1)(1+\beta) T^{1.5}} \right) = c_0 - \frac{E_{TC}}{k_B T}, \quad \text{(S62)}$$

where $c_0 = \ln(g_T N_{C0}/N_T)$. Therefore, Eq. (S62) allows us to extract the trap energy level via temperature-dependent measurement using Arrhenius-type analysis. To use this equation, we need a set of temperature-dependent measurements of slope S_0 and β from the second asymptote. An example of this analysis is presented in Fig. 2 and its corresponding discussion in the main text.

A.5 Formulas for *N*-type System

The behavior of *N*-type semiconductors mirrors that of the *P*-type equations presented in section A.1. In general, we can obtain the equations for the *N*-type materials from those of the *P*-type by using the following transformation: $H \to -H$, $\sigma_{PH} \to -\sigma_{PH}$, $\beta \to 1/\beta$, and $N_C \to N_V$. Here the minority carrier is hole, and the trap energy level is close to the valence band (VB). The trapped hole density is given as:

$$p_T = \int_0^\infty g_T(E)[1 - f_{FD}(E)]\,dE = \frac{N_T}{1 + g_T \exp[(E_{Fp} - E_T)/k_B T]}, \qquad \text{(S63)}$$

and the hole photocarrier density in the VB is:

$$\Delta p = N_V \exp[-(E_{Fp} - E_V)/k_B T], \qquad \text{(S64)}$$

where N_V is the DOS of VB (*24*):

$$N_V = 2\left(\frac{m_{d,p}^* k_B T}{2\pi\hbar^2}\right)^{3/2}, \qquad \text{(S65)}$$

with $m_{d,p}^*$ is the hole DOS effective mass. Similar to Eq. (S6), the non-linear equation relating the trapped hole and free hole is given as:

$$p_T \Delta p + g_T N_V p_T e^{-E_{TV}/k_B T} - N_T \Delta p = 0, \qquad \text{(S66)}$$

where $E_{TV} = E_T - E_V$ is the trap energy level from the valence band edge E_V (a positive number). The transport equations for the *N*-type materials can be written as:

$$\sigma = \sigma_0 + e\mu_N (p_T + (1 + 1/\beta)\Delta p) \qquad \text{(S67)}$$

$$\sigma_{PH} = -\sigma_0 - e\mu_N (p_T + (1 - 1/\beta^2)\Delta p). \qquad \text{(S68)}$$

From these equations we can solve for p_T and Δp:

$$p_T = \frac{(1-\beta)\sigma - \beta\sigma_{PH} - \sigma_0}{e\mu_N} \qquad \text{(S69)}$$

$$\Delta p = \frac{\beta^2(\sigma + \sigma_{PH})}{e\mu_N (1+\beta)}. \qquad \text{(S70)}$$

The explicit hyperbola equation in Eq. (S17) for *N*-type material becomes:

$$\sigma_{PH}(\sigma) = -\left[s_N + s_E + (2\beta - 1)\sigma + \sqrt{(s_N + s_E + \sigma)^2 - 4(s_E\sigma_0 + s_N\sigma)}\right]/2\beta, \qquad \text{(S71)}$$

where: $s_N = e\mu_0(n_0 + N_T)$ and $s_E = e g_T \mu_0 (1 + 1/\beta) N_V \exp(-\varepsilon_{TV})$.

For N_T determination, the equation for *N*-type material is the same as shown in Eq. (S46), but for E_T determination, it is given as:

$$E_{TV} = k_B T \ln\left(\frac{e\mu_0(1+1/\beta)N_V}{s_E}\right) = k_B T \ln\left(\frac{e^2 g_T \mu_0{}^2(1+\beta)N_V N_T}{\beta^3\Delta^2}\right). \quad \text{(S72)}$$

For the temperature-dependent version like in Eq. (S62), we have:

$$\ln\left(\frac{1+S_0}{(1-S_0-\beta)(1+\beta)T^{1.5}}\right) = c_0 - \frac{E_{TV}}{k_B T} \quad \text{(S73)}$$

where $c_0 = \ln(g_T N_{V0}/N_T)$.

B. Carrier-Resolved Photo-Hall Formulas with Trap

Once we solve for the trapping effect as described by the hyperbola model in Section A, we occasionally need to solve for full carrier-resolved solutions if the mobilities vary, which often occurs at high light intensity. For clarity, we revisit the CRPH solutions in the presence of trap and also address the variable mobility situation when the traps are assumed to be full ($n_T \sim N_T$). They are largely similar to our earlier CRPH model (*21*), except we include the role of n_T in the solutions.

Here, we attempt to solve for three uknowns: $\Delta p, \mu_P$ and μ_N from three experimentally measured quantities: σ, H, and $\Delta\mu = d(\sigma^2 H)/d\sigma$. We consider the hole and electron density: $p = p_0 + \Delta p$, $n = n_0 + \Delta n \simeq \Delta n$, and the charge neutrality condition: $\Delta p = \Delta n + n_T$. Starting from the bipolar Hall transport equations in Eqs. (S7) and (S8), we have:

$$\sigma = e[(p_0+\Delta p)\mu_P + (\Delta p - n_T)(\mu_P - \Delta\mu)] \quad \text{(S74)}$$

$$\sigma^2 H = e[\mu_P{}^2(p_0+\Delta p) - (\mu_P-\Delta\mu)^2(\Delta p - n_T)]. \quad \text{(S75)}$$

Solving for $\boldsymbol{\mu_P}$ and $\boldsymbol{\Delta p}$, we have:

$$\mu_P = \frac{1}{2}\left[\Delta\mu - \sqrt{\frac{4\sigma(\sigma H - \Delta\mu) + e\Delta\mu^2(n_T+p_0)}{e(p_0+n_T)}}\right] \quad \text{(S76)}$$

$$\Delta p = \frac{\sigma + e n_T(\mu_P - \Delta\mu) - p_0\mu_P e}{e(2\mu_P - \Delta\mu)}. \quad \text{(S77)}$$

Then we can obtain the full solutions: $\mu_N = \mu_P - \Delta\mu$ and $\Delta n = \Delta p - n_T$. For the calculation when the trap is full (e.g. at high light intensity), we can use: $n_T \sim N_T$ and thus $\Delta n = \Delta p - N_T$.

As explained in our previous work (Ref. (*21*), SI section B.2), if the mobilities vary – which could occur at high injection ($\Delta p, \Delta n \gg p_0$) – the actual mobility difference will be modified. We rederive again these results and simplify them:

$$\Delta\mu = \left[\frac{d(\sigma^2 H)}{d\sigma} + c_1\right]c_2, \quad \text{where:} \quad \text{(S78)}$$

$$c_1 = 2e\left(n\,\mu_N \frac{d\mu_N}{d\sigma} - p\mu_P \frac{d\mu_P}{d\sigma}\right) \tag{S79}$$

$$c_2 = \left(1 - e\left[n\frac{d\,\mu_N}{d\sigma} + p\frac{d\,\mu_P}{d\sigma}\right]\right)^{-1}. \tag{S80}$$

We note that if the mobilities are constant, Eq. (S78) reduces to the basic form: $\Delta\mu = d(\sigma^2 H)/d\sigma$ as expected. If the initial solution of the CTRPH exhibits significant variation in the mobilities then we need to employ this "variable-mobility correction" in iterative fashion, until the solution converges (*21*). The correction tends to increase $\Delta\mu$ at high light intensity, as we found in the perovskite calculation in Fig. S7a.

C. Summary of Carrier and Trap Parameter Extraction using CTRPH Technique

Here we summarize all the parameters that can be calculated from the CTRPH technique in two lists: charge carrier parameters and trap parameters. These include nearly all properties that we want to know from charge carriers and trap in semiconductor. The entries in yellow mark the primary parameters, that come as direct results from the experimental measurements. The rest are secondary parameters which mean they can be derived from the primary parameters. In general, the key data in the CTRPH experiment is a set of σ, H and G_γ measurements at various light intensities, and they can be repeated as a function of temperature T. Our theoretical model, as described in section A, could yield all the parameters below for both P and N-type semiconductors (with some slight differences in the formulas between the two).

C.1 Charge carrier parameters:

No	Parameter	Description	Formula for *P* or *N*-type system	
**1.**	Δn	electron photocarrier density	P	$\Delta n = \dfrac{\sigma - \sigma_{PH}}{e\mu_P \beta(\beta+1)}$
			N	$\Delta n = \Delta p + p_T$
**2.**	Δp	hole photocarrier density	P	$\Delta p = \Delta n + n_T$
			N	$\Delta p = \dfrac{\beta^2(\sigma + \sigma_{PH})}{e\mu_N(1+\beta)}$
**3.**	n_T or p_T	trapped electron or hole density	P	$n_T = \dfrac{(\beta-1)\sigma + \sigma_{PH} - \beta\sigma_0}{e\mu_P\beta}$
			N	$p_T = \dfrac{(1-\beta)\sigma - \beta\sigma_{PH} - \sigma_0}{e\mu_N}$

**4.**	μ_N	electron mobility	P	$\mu_N = \beta \mu_P$
			N	$\mu_N = \mu_0$ (dark)
**5.**	μ_P	hole mobility	P	$\mu_P = \mu_0$ (dark)
			N	$\mu_P = \mu_N / \beta$
**6.**	τ_N	electron recombination lifetime	$\tau_N = \Delta n / G$	
**7.**	τ_P	hole recombination lifetime	$\tau_P = \Delta p / G$	
**8.**	D_N	electron diffusion coefficient	$D_N = k_B T \mu_N / e$	
**9.**	D_P	hole diffusion coefficient	$D_P = k_B T \mu_P / e$	
**10.**	D_A	ambipolar diffusion coefficient	$D_A = \dfrac{n+p}{n / D_P + p / D_N}$	
**11.**	$L_{D,N}$	electron diffusion length	$L_{D,N} = \sqrt{D_N \tau_N}$	
**12.**	$L_{D,P}$	hole diffusion length	$L_{D,P} = \sqrt{D_P \tau_P}$	
**13.**	$L_{D,A}$	ambipolar diffusion length	$L_{D,A} = \sqrt{D_A \tau_A}$	
**14.**	QF_N	electron quasi-Fermi level	$QF_N = k_B T \ln(n / n_i)$	
**15.**	QF_P	hole quasi-Fermi level	$QF_P = k_B T \ln(p / n_i)$	
**16.**	$QFLS$	quasi-Fermi level splitting	$QFLS = QF_N + QF_P$	
**17.**	η	ideality factor	$\eta = (dQFLS / d \ln G_\gamma) / k_B T$	

Table S1. The charge carrier parameters extracted using CTRPH method. Parameters in yellow indicate primary extraction results.

Notes:

(1) In the CTRPH model (entry #1-5), we assume constant mobilities (and thus β) and constant dark carrier density p_0 or n_0, which implies there should not be any persistent photoconductivity effect (see e.g. Ref. (*40*)). At high light intensity the mobilities could vary, and one could use variable mobility model as described in previous work (*21*) and section B.

(2) For lifetime calculation (#6 and 7) we assume 100% quantum efficiency thus generation rate G and the absorbed photon density G_γ are equal: $G = G_\gamma$. We also assume 100% exciton dissociation efficiency.

(3) For ambipolar diffusion length (#13), we can calculate the effective lifetime as: $\tau_A = (n+p)/(n/\tau_N + p/\tau_P)$, using Matthiessen's rule of carrier lifetimes weighted by their respective densities.

(4) Given electron and hole densities, the quasi Fermi levels and the quasi Fermi level splitting (QFLS) can also be calculated as suggested in Ref. (*20*) and shown in entry #14, 15 and 16. n_i is the intrisinc carrier density (*24*). The quasi Fermi level is calculated with respect to the intrinsic Fermi level E_i. The QFLS is useful to estimate the potential open circuit voltage that a material could deliver in a solar cell application (*41*).

(5) The ideality factor η can also be calculated (*20*), which is obtained from general relationship that relates QFLS with photon flux Φ (*42*): $\Phi = \Phi_0 \exp(QFLS / \eta k_B T)$. Since Φ is proportional to the absorbed photon density G_γ, we can derive η as shown in entry #17.

(6) We can also analyze the carrier density as a power law of light intensity, i.e.: $\Delta n = G_\gamma^m$. Evaluating the exponent factor m is useful to analyze the recombination mechanisms (see e.g. (*34, 43*)). At high light intensiity limit, where $\Delta n \simeq \Delta p$, the exponent factor m is related to the ideality factor η as: $m = \eta / 2$. This can be derived as follows:

$$QFLS = k_B T \ln(\Delta n^2 / n_i^2), \qquad dQFLS / d\ln\Delta n = 2 k_B T \tag{S81}$$

$$\eta = \frac{dQFLS}{d\ln G_\gamma}\frac{1}{k_B T} = \frac{dQFLS}{d\ln\Delta n}\frac{d\ln\Delta n}{d\ln G_\gamma}\frac{1}{k_B T} = 2m. \tag{S82}$$

For example, many high performance solar cells has $\eta \sim 1$ (*44*), which implies: $m \sim 0.5$ (at high light intensity) as we observe in our perovskite study (Fig. 3 and Ref. (*21*)).

C.2 Trap parameters:

No	Parameters	Description		Formula
**1.**	N_T	trap density		$\tilde{N}_T = \dfrac{\sigma_1 - \sigma_0}{e\mu_0}$
**2.**	E_T	trap energy level	P	$E_{TC} = k_B T \ln\left(\dfrac{e^2 g_T \mu_0^2 \beta^2 (\beta+1) N_C N_T}{\Delta^2}\right)$<br>$\ln\left(\dfrac{1-S_0}{(\beta+S_0-1)(1+\beta)T^{1.5}}\right) = c_0 - \dfrac{E_{TC}}{k_B T}$
			N	$E_{TV} = k_B T \ln\left(\dfrac{e^2 g_T \mu_0^2 (1+\beta) N_V N_T}{\beta^3 \Delta^2}\right)$<br>$\ln\left(\dfrac{1+S_0}{(1-S_0-\beta)(1+\beta)T^{1.5}}\right) = c_0 - \dfrac{E_{TV}}{k_B T}$
**3.**	σ_N	electron scattering cross section	P	$\sigma_N = 1/v_{Tn} N_T \tau_{N,SRH}$
			N	$\sigma_N = 1/v_{Tn} n_T \tau_{N,SRH}$
**4.**	σ_P	hole scattering cross section	P	$\sigma_P = 1/v_{Tp} n_T \tau_{P,SRH}$
			N	$\sigma_P = 1/v_{Tp} N_T \tau_{P,SRH}$

Table S2. The trap parameters extracted using CTRPH method. Parameters in yellow indicate primary extraction results.

Note:

(1) For best N_T and E_T extraction, trap detection can be obtained when the $\sigma_{PH} - \sigma$ bending is significant, i.e., the minority mobility is larger than that of majority and sufficiently low temperature measurement ($E_{TC} \gg k_B T$).

(2) For scattering cross section calculation (#3 and 4), we use Schottky-Read-Hall (SRH) recombination model (*45*). Considering a *P*-type material as an example, we have the rate equations where SRH process dominate:

$$dn/dt = G - n\sigma_N v_{Tn}(N_T - n_T) \tag{S83}$$

$$dp/dt = G - p\sigma_P v_{Tp} n_T \,. \tag{S84}$$

At very low light intensity where the trap is nearly empty ($n_T \ll N_T$) we have:

$$\tau_{N,SRH} = \frac{\Delta n}{G} = \frac{1}{\sigma_N v_{Tn} N_T} \quad \text{or} \quad \sigma_N = \frac{1}{v_{Tn} N_T \tau_{N,SRH}} \tag{S85}$$

$$\tau_{P,SRH} = \frac{\Delta p}{G} = \frac{1}{\sigma_P v_{Tp} n_T} \quad \text{or} \quad \sigma_P = \frac{1}{v_{Tp} n_T \tau_{P,SRH}}, \tag{S86}$$

where $v_T = \sqrt{8k_B T/\pi m^*}$ is the thermal velocity of electron or hole and m* is their corresponding effective mass (*46*). Note that, using example of *P*-type system, for the σ_P calculation, since n_T may be inaccurate at low light intensity, we can use the rate calculation: $1/\tau_P$ vs. n_T, i.e., $\sigma_P = [d(1/\tau_P)/dn_T]/v_{Tp}$.

D. Examples of Carrier and Trap Resolved Photo-Hall Effect Analysis

D.1 Optical and physical properties of the test samples

No	Parameter	*Symbol*	Unit	Sample		
				***p*-SOI**	***n*-Si**	**$FAPbI_3$**
	**Sample:**					
1.	Thickness	d	μm	5.0	280	0.7
2.	Active area (width x length)	$W \times L$	mm	1 x 2	2 x 4	2 x 4
3.	Reflectivity	R		0.334	0.352	0.171
4.	Absorption coefficient	α	/cm	3.72×10^3	3.66×10^3	4.43×10^3
5.	DOS effective mass	m_e^*/m_h^*		1.08/0.55	1.08/0.55	0.184/0.213
6.	Intrinsic carrier density	n_i	$/cm^3$	1.1×10^{10}	1.1×10^{10}	1.57×10^5
	**PDL Hall System:**					
7.	Light wavelength	λ	μm	615	615	615
8.	Reference PD QE	QE_{REF}		0.765	0.765	0.765

9.	PD calibration factor	k_{PD}		0.70	0.62	0.57
10.	Optical setup constant	k_G	/Acm^{3}s	8.58x10^{22}	1.56x10^{21}	6.77x10^{23}

Table S3. The device and optical parameters for the samples used in this work. PD is photodetector and QE is quantum efficiency. The $FAPbI_3$ perovskite effective mass is obtained from Ref. (*47*).

We present the physical, optical and system parameters for all three samples that we used in this work as shown in Table S3 above.

Notes:

(1) Sample active area is the area defined by the four Hall bar terminals that are used in the longitudinal and transverse resistance measurement.

(2) The effective mass is used for effective DOS calculation (N_C and N_V), intrinsic carrier density (n_i) for quasi-Fermi levels calculation and thermal velocity (v_T) for the trap scattering cross section.

(3) In the experiment, we monitor the light intensity by measuring the current in the monitor photodetector cell ($I_{PD,MON}$) (see Fig. 1a). The calculation detail is also presented in our previous work (Ref. (*21*), SM section C). The optical properties of the films are needed to calculate the absorbed photon density given as: $G_\gamma = k_G(\lambda) I_{PD,MON}$, where k_G is the optical setup constant that is wavelength-dependent:

$$k_G(\lambda) = \frac{k_{PD}\left[1-R(\lambda)\right]}{e\, QE_{REF}(\lambda)\, A_{REF}} \frac{[1-\exp(-\alpha(\lambda)\, d)]}{d}, \tag{S87}$$

where k_{PD} is the light calibration factor as measured by photodetector (PD), i.e., the ratio between the reference and monitor PD: $k_{PD} = I_{PD,REF}/I_{PD,MON}$, averaged throughout all operating light intensity.

(4) The light intensity (in W/m^2) impinging on the sample is:

$$I_L = \frac{I_{PD,MON} k_{PD}}{QE_{REF} A_{REF}} \frac{h\,c}{e\,\lambda}. \tag{S88}$$

where A_{REF} =7.5 mm^2 is the area of the reference PD, h is the Planck constant, and c is the speed of light. This formula is used to calculate the maximum light intensity impinging on the sample (e.g. see Fig. 1 and 3).

D.2 Carrier and Trap Resolved Photo-Hall Analysis of *p*-SOI sample

Here we present the complete CTRPH analysis of the *p*-SOI sample that is discussed in the main text. The physical and optical parameters of this sample are shown in Table S3. First, we obtain the photo-Hall data and perform the curve fitting as discussed in the main text and presented again in Fig. S5a. From the dark Hall data point, we have: σ= 3.98 S/m, p_0 = 5.65×10^{14} /cm^3 and μ_P =

440 cm^2/Vs. The curve-fitting using the hyperbolic model in Eq. (S21) yields: $\beta = 3.26$, N_T = 1.54×10^{14} /cm^3 and E_T = 0.46 eV. Here we obtain the electron mobility: $\mu_N = \beta\mu_P$ =1432 cm^2/Vs which is close to the known values of mobilities in silicon (1500 cm^2/Vs (*1*)).

We use Eq. (S3), (S4) and (S21) to calculate Δn, n_T and thus obtain $n = \Delta n$ and $p = \Delta n + n_T$ vs. G_γ as shown in Fig. S5b. Unlike in our earlier work, where we neglect the trapping effect, now we can completely resolve the electron and hole properties and thus calculate the mobility, lifetime and diffusion lengths for both as shown in Fig. S5b-g. We also report the quasi-Fermi levels and its ideality factor. In addition, using the formulas in Table S2, we can calculate the scattering cross section factors which are: σ_N=1.81×10^{-2} nm^2 and σ_P =3.72×10^{-3} nm^2 for electron and hole respectively.

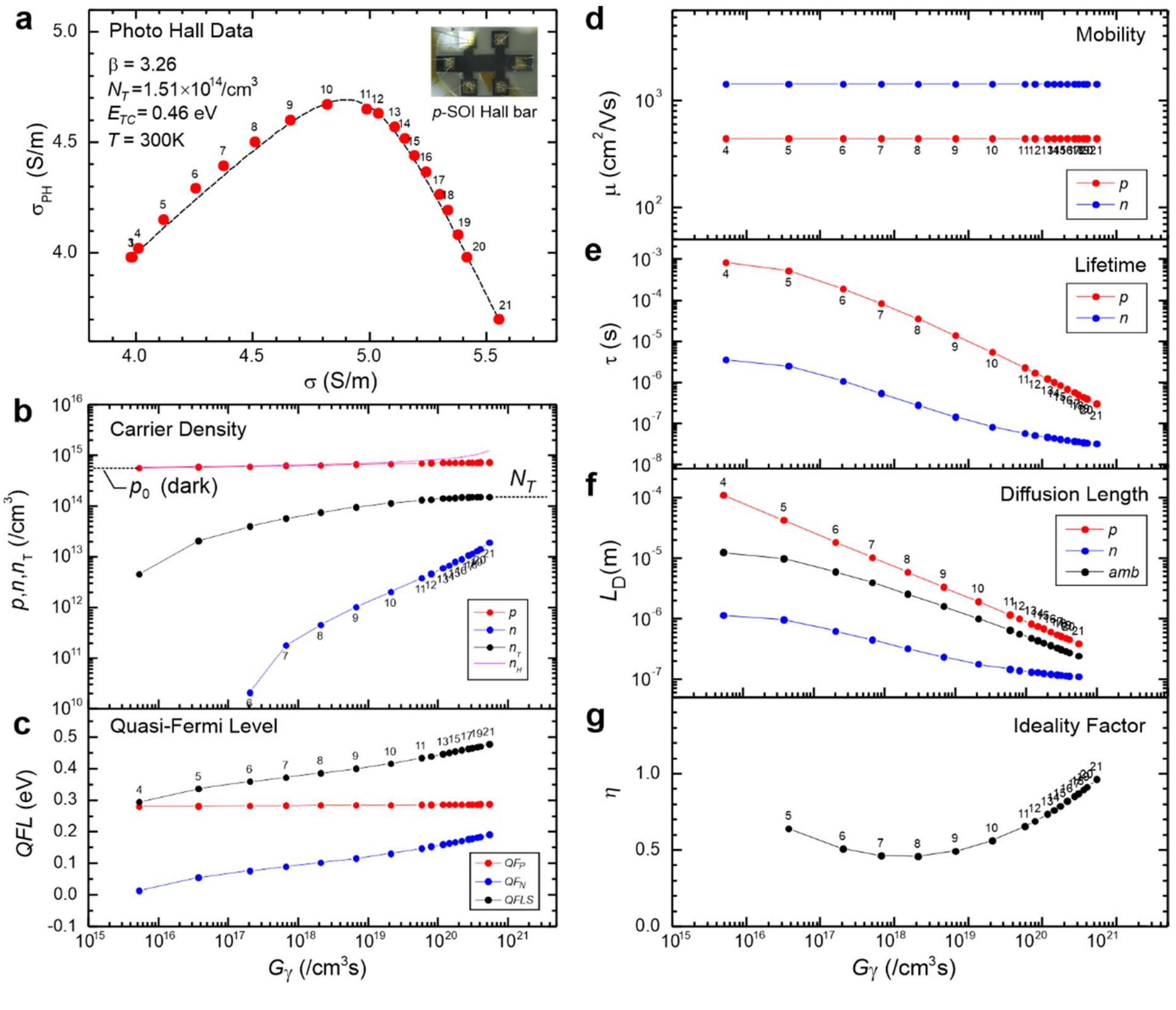

Figure S5. Complete CRPH analysis of the *p*-SOI sample: (a) The photo Hall data with laser light λ= 615 nm and maximum intensity I_L =157 mW/cm^2. I**nset:** the *p*-SOI sample. **(b)** Carrier density for p, n and n_T vs. absorbed photo density G_γ, and $n_H = 1/eH$ is the Hall density. **(c)** Quasi Fermi level. **(d)** Mobility. **(e)** Recombination lifetime. **(f)** Diffusion length. **(g)** Ideality factor.

D.3 Carrier and Trap Resolved Photo-Hall Analysis of n-Si sample

We present the CTRPH study in an *N*-type single crystal silicon as shown in Fig. S6. The physical and optical properties of the sample are shown in Table S3. From the dark Hall data point, we have: σ_0= 0.0982 S/m, dark electron density: n_0 = 3.58×10^{12} /cm^3, and mobility μ_N = 1703 cm^2/Vs. We plot the photo Hall data: σ_{PH} vs. σ as shown in Fig. S6a. We observe the plot exhibits a bending behavior expected for an *N*-type system with $\mu_P < \mu_N$, i.e., the quadrant III behavior in Fig. 1d, where there are two line segments with negative slopes and a weak bending. We determine the inflection point at σ_1 = 0.167 S/m, and using Eq. (S3), we obtain trap density N_T = 2.49×10^{12} /cm^3.

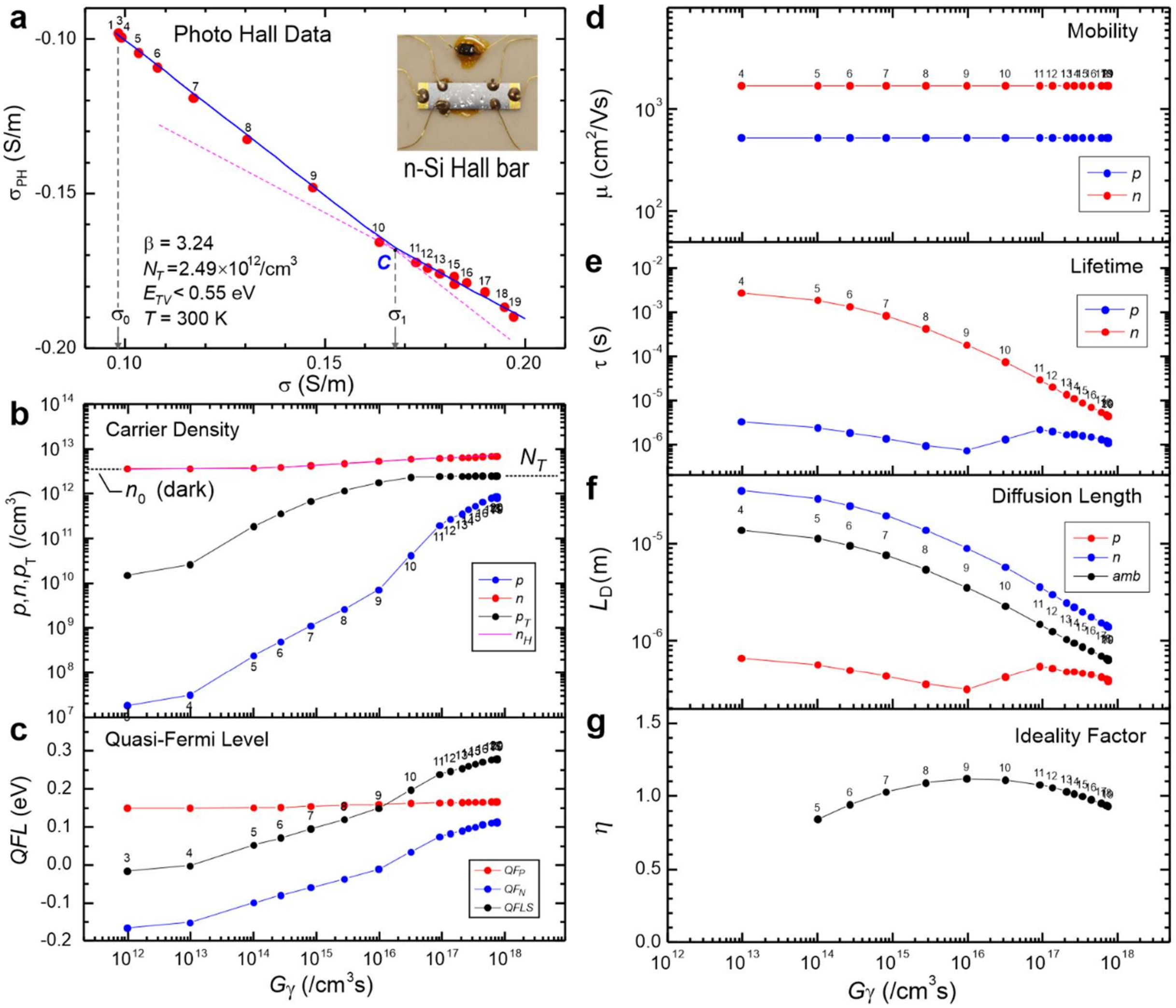

Figure S6. Complete CRPH analysis of an *N*-type Si sample: (a) The photo Hall data with laser light λ= 615 nm and maximum intensity I_L =10.5 mW/cm^2. Blue curve: hyperbola fit from Eq. (S21). **Inset:** the *N*-type Si Hall sample. **(b)** Carrier density vs. absorbed photo density (G_γ). **(c)** Quasi-Fermi levels. **(c)** Mobility. **(d)** Recombination lifetime. **(e)** Diffusion length. **(g)** Ideality factor.

As mentioned in the main text, we obtain the slope: $S = -1 + 1/\beta$ =0.691, which yields β = 3.24 and thus hole (minority) mobility of: $\mu_P = \mu_N/\beta$ = 526 cm^2/Vs. For the trap energy level, due to very weak bending near the inflection point and insufficient data points, we can not determine the parameter Δ accurately. However we can establish its upper limit, i.e., $\Delta < 9.4\times10^{-4}$ S/m and thus upper bound of the trap energy, i.e., $E_{TV} < 0.55$ eV.

The full set of CTRPH analysis plots including carrier density, quasi-Fermi levels, mobility, lifetime, diffusion lengths and ideality factors are presented in Fig. S6b-g. Furthermore, from the lifetime data near dark, we obtain scattering cross section: $\sigma_N = 1.1\times10^{-2}$ nm^2 and $\sigma_P = 1.49\times10^{-3}$ nm^2 for electron and hole respectively.

D.4 Carrier and Trap Resolved Photo-Hall Analysis of the $FAPbI_3$ Perovskite Sample

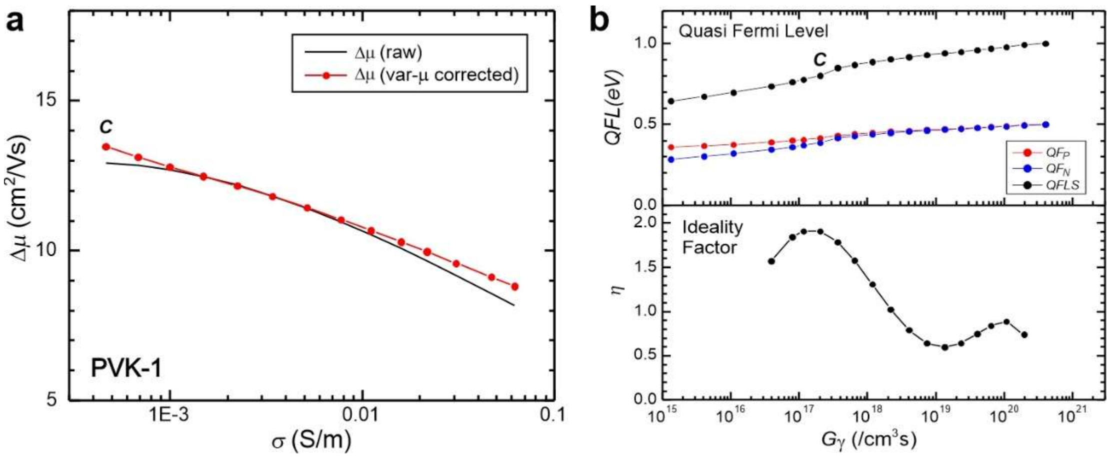

Figure S7. Additional analysis on the $FAPbI_3$ perovskite sample "PVK-1": (a) The mobility difference ($\Delta\mu$) before and after variable-mobility correction. **(b)** Quasi Fermi level and ideality factor as a function of absorbed photon density G_γ.

We present the details of the $FAPbI_3$ perovskite CTRPH analysis sample "PVK-1". The initial objective is to obtain a set of primary quantities: μ_P, μ_N, Δn, n_T, and Δp. As mentioned in the text, we divide the analysis to two regimes: (1) Trapping regime (segment D-C-E) and (2) Trap-full or high light intensity regime (segment E-F). In the first regime, the $\sigma_{PH} - \sigma$ plot shows a weak bending, consistent with the quadrant II behavior in Fig. 1d, where the majority mobility is larger than that of minority ($\mu_P > \mu_N$). Fortunately, we could observe an inflection point at C, which allows us to extract the trap density using Eq. (S3), i.e., $N_T = 1.9\times10^{12}$ /cm^3. The segment C-E of the curve, where the trap is expected to be full, yields a slope S = 0.81 and thus $\beta \sim 0.19$. The mobility in the dark is $\mu_P \sim (12 \pm 2)$ cm^2/Vs. As the bending is weak, we cannot reliably determine the gap "Δ", however using simulation of the hyperbola equation in Eq. (S21), we estimate $E_T \sim$

0.3-0.7 eV. Then we can calculate Δn, n_T and Δp using equations in Table S1 and plotted in Fig. 3b.

Next, we analyze the second regime at high light intensity. To solve the photo-Hall transport problem, we first calculate the mobility difference using: $\Delta\mu = (2 + d\ ln\, H\ /d\ ln\, \sigma)\sigma H,$ which is more appropriate to perform at high injection regime like in this perovskite sample (*21*). To facilitate smooth derivative calculation (for $\Delta\mu$ and variable-mobility correction), we fit the H vs. σ data with a smooth second-degree polynomial in the log-log scale. To ensure continuous solution, we start the analysis from point C, where the trap is approximately full. We obtain the (raw) $\Delta\mu$ data which is not constant as shown in Fig. S7a. Then we apply the variable mobility correction using Eq. (S78) in iterative fashion until we obtain self-consistent $\Delta\mu$ solution as shown in Fig. S7b (red curve). The increase in mobility at high light intensity could be due to several factors such as enhanced screening of Coulombic interaction between carriers and charged defects which reduces scattering, lowering of intergrain potential barrier for polycrystalline material (Ref. (*48*), Ch. 2) and trap that are full thus reducing its harmful effects. The ideality factor (η) plot in Fig. S7b also shows the expected trend, it starts around 2 while the trapping effect dominates and drops to ~ 1 when the trap is full.

Finally, we solve for: Δn, n_T and Δp using Eqs. (S76) and (S77) and other charge carrier parameters as shown in Fig. 3. In addition, we also present the Quasi-Fermi Level plots and its ideality factor in Fig. S7b. We note that, at maximum light intensity (~16 mW/cm^2), the $QFLS$ is ~1.0 V which is close to V_{OC} ~ 1.12 V of the corresponding solar cell at the same intensity (Ref. (*28*), Fig. 2, "target cell"). Finally, from the lifetime data near dark, we obtain scattering cross section: σ_N = 0.35 nm^2 and σ_P = 0.64 nm^2 for electron and hole respectively. We also make another comment, in the high light intensity regime E-F (Fig. 3b), we observe: $\Delta n \simeq \Delta p$, thus one could also solve the problem while ignoring the trapping effect like in our previous report (*21*).

Temperature-dependent data in perovskite:

We have also performed temperature-dependent study on the same perovskite sample "PVK-1" from T = 260K to 340 K and focus on trap detection from the bending of σ_{PH} vs. σ curve. As discussed in the main text, the bending behavior in this perovskite is generally weak following quadrant II (Fig. 1d) behavior. Strictly speaking, the σ_{PH} vs. σ hyperbola is defined in linear scale, but to identify the inflection point – marked by a sudden change in slope (or occasionally appearing as "peak") – it is often more apparent to observe it in a log-log plot.

In general, we observe behavior similar to that of the *p*-SOI sample (Fig. 2), namely, the curve bending is more pronounced at lower temperatures such as at T = 260 K. At higher temperatures, e.g. at T=320 K, the bending becomes much weaker, making it necessary to confirm the bending in the log-log plot of H vs. σ (Fig. S8d). Nevertheless, we are able to extract the trap density from these curves with average N_T = (6.2±1.5)×10^{12} /cm^3, as summarized in Table S4 below.

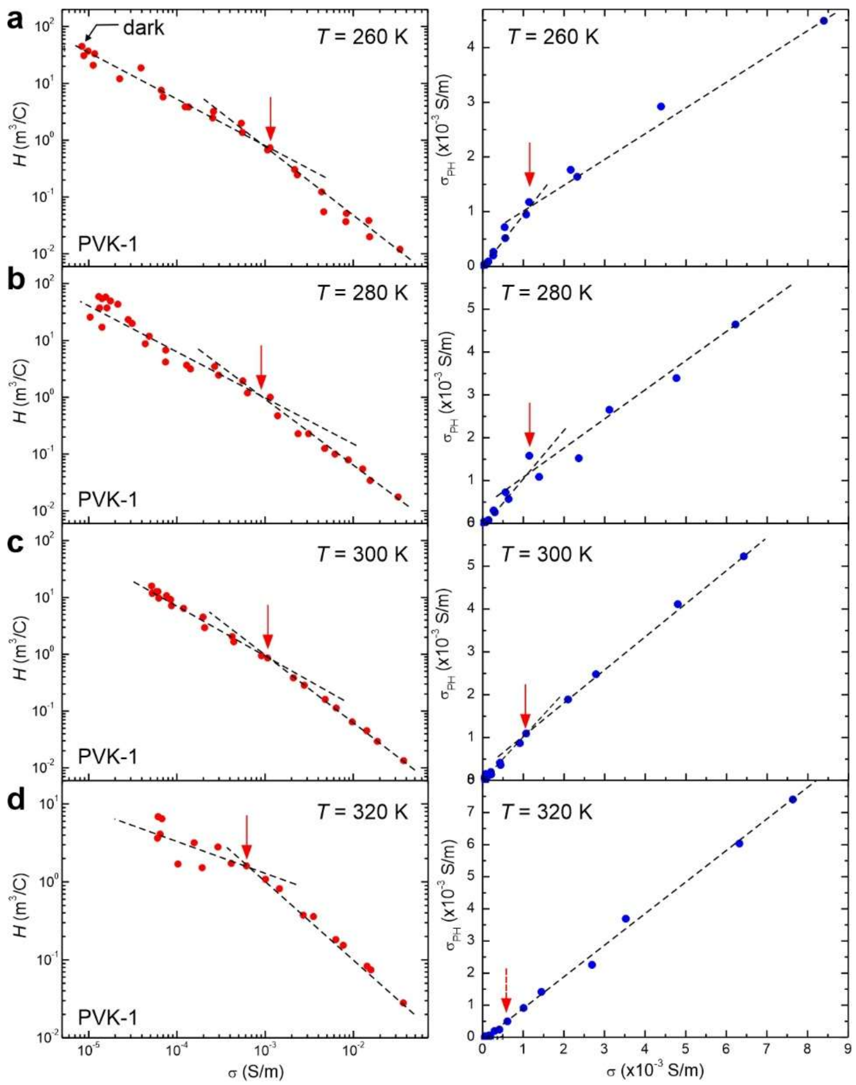

Figure S8. Variable temperature photo-Hall data on the $FAPbI_3$ sample "PVK-1" (T = 260 K to 320 K). The plots are shown as H vs. σ plot (in log-log scale) and σ_{PH} vs. σ (in linear scale).

Additional measurement in perovskite:

To further verify the trap detection technique in perovskite we have repeated the photo Hall measurement in another sample ("PVK-2") from a batch of similar fabrication process as shown in Fig. S9. We observe similar H vs. σ data with bending behavior in the σ_{PH} vs. σ plot that yields trap density $N_T = 9.6\times10^{12}$ /cm^3, close to that of sample PVK-1. We note that as the H vs. σ data is similar to that of the "PVK-1" sample, thus this sample also yields similar set of analysis results as shown in Fig. 3 in the main text.

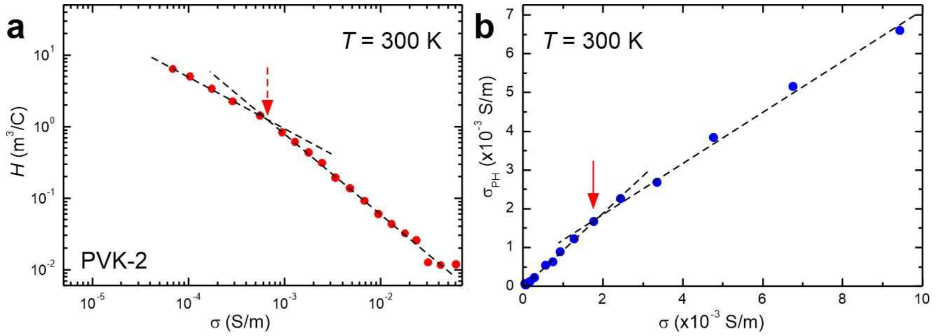

Figure S9. Photo Hall data from another $FAPbI_3$ perovskite sample "PVK-2". The plots show similar bending behavior in: **(a)** *H* vs. σ plot (in log-log scale), and **(b)** σ_{PH} vs. σ plot (in linear scale).

Sample	Temperature	Majority mobility (dark)	Carrier density (dark)	Conductivity (dark)	Conductivity (inflection point)	Trap Density
	T	μ_P	p_0	σ_0	σ_1	N_T
	(K)	(cm^2/Vs)	(/cm^3)	(S/m)	(S/m)	(/cm^3)
**PVK-1**	260	10.6	6.31E+10	1.07E-05	1.07E-03	6.3E+12
	280	9.1	1.24E+11	1.80E-05	1.20E-03	8.1E+12
	300	8.9	3.65E+11	5.20E-05	8.90E-04	5.9E+12
	320	7.8	4.90E+11	6.13E-05	6.16E-04	4.4E+12
**PVK-2**	300	11.2	2.74E+11	4.89E-05	1.76E-03	9.6E+12

Table S4. Trap detection summary from sample perovskite samples PVK-1 and PVK-2.

E. Summary of Major Trap Detection Techniques

No.	Technique	Trap properties			Carrier* properties	Notes
		N_T	E_T	$\sigma_{P,N}$		
1.	Deep-Level Transient Spectroscopy (DLTS) (*8, 49*)	√	√	√	X	Need junction. Could detect multiple trap levels, energy profile, has high sensitivity.
2.	Drive-Level Capacitance Profiling (DLCP) (*11, 50*)	√	√	X	X	Need junction. Could yield trap spatial information.

3.	Space Charge Limited Current (SCLC) (*13, 51, 52*)	√	X	X	μ	Can detect hole or electron trap density. Drift majority mobility can be extracted.
4.	Transient photo-luminescence (TrPL) (*16*)	√	X	√	τ	Requires modeling to extract trap density N_T and Δn.
5.	Thermal Admittance Spectroscopy (TAS) (*14, 15*)	√	√	√	X	Need junction. Yield energy profile.
6.	Thermally Stimulated Current (TSC) (*53, 54*)	√	√	X	X	May fail detect traps due to incomplete trap filling and partial detrapping during thermalization. N_T can only be estimated.
7.	Transient Photo-conductivity (TRPC) or Microwave Conductivity (TRMC) (*17, 55*)	√	X	√	$\mu_P+\mu_N$, τ	Modeling required to extract N_T.
8.	Transient Photo Hall spectroscopy (*19*)	√	√	√	τ	Need to have high mobility to yield strong transient signal. More carrier properties can be extracted but not attempted.
9.	Photo Hall effect spectroscopy (*18*)	X	√	√	X	Sub-bandgap light needed. $\sigma_{N/P}$ measured by complementary transient photoconductivity. More carrier properties can be extracted but not attempted.
10.	Constant Light-Induced Magneto Transport/Photo Hall (CLIMAT) (*20*)	√	~	√	Δn, Δp, n_T, μ_N, μ_P, τ_N, τ_P	N_T is determined from simulation and fitting. E_T is estimated. Yield nearly all carrier-resolved charge carrier parameters.
11.	Carrier and Trap Resolved Photo Hall effect (CTRPH/ this work)	√	√	√	Δn, Δp, n_T, μ_N, μ_P, τ_N, τ_P	N_T and E_T are determined from exact formulas, including *T*-dependent equation for E_T. Yield nearly all carrier-resolved charge carrier parameters.

Table S5. Major trap detection techniques and their output capability. $\sigma_{P/N}$ is the capture cross section for hole or electron. Marker √, ~, and × indicate capable to measure, approximate and not capable respectively. *Only primary carrier properties outputs are listed. There could be more secondary carrier parameters that can be calculated (e.g. D, L_D and quasi-Fermi levels), see section C for a complete list.

Table S5 summarizes various major trap detection techniques and their output capabilities, including some comments. The cited references are mostly application examples in perovskite materials with dominant techniques that include capacitance-based methods (DLCP, DLTS) and trap-filled limit (SCLC) methods. However, those techniques are limited by rather high detection threshold for thin film devices as they are essentially charge-based detection methods (*7*). The CTRPH technique yields the most comprehensive list of charge and carrier trap parameters output as much as $17\times N$ charge carrier and 4 trap parameters in total, with N is the number of light intensity settings. Best results are obtained when the $\sigma_{PH} - \sigma$ plot has strong hyperbolic curvature (low eccentricity).

REFERENCES

1. D. K. Schroder, “Ch. 5 Defects” in *Semiconductor Material and Device Characterization* (Wiley, ed. 3, 2005), p. 251, 504.

2. J. Jeong, M. Kim, J. Seo, H. Lu, P. Ahlawat, A. Mishra, Y. Yang, M. A. Hope, F. T. Eickemeyer, M. Kim, Y. J. Yoon, I. W. Choi, B. P. Darwich, S. J. Choi, Y. Jo, J. H. Lee, B. Walker, S. M. Zakeeruddin, L. Emsley, U. Rothlisberger, A. Hagfeldt, D. S. Kim, M. Grätzel, J. Y. Kim, Pseudo-halide anion engineering for α-$FAPbI_3$ perovskite solar cells. *Nature* **592**, 381–385 (2021).

3. J. Park, J. Kim, H.-S. Yun, M. J. Paik, E. Noh, H. J. Mun, M. G. Kim, T. J. Shin, S. I. Seok, Controlled growth of perovskite layers with volatile alkylammonium chlorides. *Nature* **616**, 724–730 (2023).

4. H. Chen, C. Liu, J. Xu, A. Maxwell, W. Zhou, Y. Yang, Q. Zhou, A. S. R. Bati, H. Wan, Z. Wang, L. Zeng, J. Wang, P. Serles, Y. Liu, S. Teale, Y. Liu, M. I. Saidaminov, M. Li, N. Rolston, S. Hoogland, T. Filleter, M. G. Kanatzidis, B. Chen, Z. Ning, E. H. Sargent, Improved charge extraction in inverted perovskite solar cells with dual-site-binding ligands. *Science* **384**, 189–193 (2024).

5. M. A. Green, E. D. Dunlop, M. Yoshita, N. Kopidakis, K. Bothe, G. Siefer, D. Hinken, M. Rauer, J. Hohl-Ebinger, X. Hao, Solar cell efficiency tables (Version 64). *Prog. Photovolt. Res. Appl.* **32**, 425–441 (2024).

6. H. Jin, E. Debroye, M. Keshavarz, I. G. Scheblykin, M. B. J. Roeffaers, J. Hofkens, J. A. Steele, It’s a trap! On the nature of localised states and charge trapping in lead halide perovskites. *Mat. Hor.* **7**, 397–410 (2020).

7. J. Siekmann, S. Ravishankar, T. Kirchartz, Apparent defect densities in halide perovskite thin films and single crystals. *ACS Energy Lett.* **6**, 3244–3251 (2021).

8. D. Lang, Deep-level transient spectroscopy: A new method to characterize traps in semiconductors. *J. Appl. Phys.* **45**, 3023–3032 (1974).

9. X. Ren, B. Zhang, L. Zhang, J. Wen, B. Che, D. Bai, J. You, T. Chen, S. Liu, Deep-level transient spectroscopy for effective passivator selection in perovskite solar cells to attain high efficiency over 23%. *ChemSusChem* **14**, 3182–3189 (2021).

10. C. Michelson, A. Gelatos, J. Cohen, Drive-level capacitance profiling: Its application to determining gap state densities in hydrogenated amorphous silicon films. *Appl. Phys. Lett.* **47**, 412–414 (1985).

11. Z. Ni, C. Bao, Y. Liu, Q. Jiang, W. Q. Wu, S. Chen, X. Dai, B. Chen, B. Hartweg, Z. Yu, Z. Holman, J. Huang, Resolving spatial and energetic distributions of trap states in metal halide perovskite solar cells. *Science* **367**, 1352–1358 (2020).

12. R. H. Bube, Trap density determination by space-charge-limited currents. *J. Appl. Phys.* **33**, 1733–1737 (1962).

13. E. A. Duijnstee, J. M. Ball, V. M. Le Corre, L. J. A. Koster, H. J. Snaith, J. Lim, Toward understanding space-charge limited current measurements on metal halide perovskites. *ACS Energy Lett.* **5**, 376–384 (2020).

14. H.-S. Duan, H. Zhou, Q. Chen, P. Sun, S. Luo, T.-B. Song, B. Bob, Y. Yang, The identification and characterization of defect states in hybrid organic–inorganic perovskite photovoltaics. *Phys. Chem. Chem. Phys.* **17**, 112–116 (2015).

15. S. Wang, P. Kaienburg, B. Klingebiel, D. Schillings, T. Kirchartz, Understanding thermal admittance spectroscopy in low-mobility semiconductors. *J. Phys. Chem. C* **122**, 9795–9803 (2018).

16. S. D. Stranks, V. M. Burlakov, T. Leijtens, J. M. Ball, A. Goriely, H. J. Snaith, Recombination kinetics in organic-inorganic perovskites: Excitons, free charge, and subgap states. *Phys. Rev. Appl.* **2**, 034007 (2014).

17. E. M. Hutter, G. E. Eperon, S. D. Stranks, T. J. Savenije, Charge carriers in planar and meso-structured organic-inorganic perovskites: Mobilities, lifetimes, and concentrations of trap states. *J. Phys. Chem. Lett.* **6**, 3082–3090 (2015).

18. A. Musiienko, P. Moravec, R. Grill, P. Praus, I. Vasylchenko, J. Pekarek, J. Tisdale, K. Ridzónová, E. Belas, L. Landová, B. Hu, E. Lukosi, M. Ahmadi, Deep levels, charge transport and mixed conductivity in organometallic halide perovskites. *Energ. Environ. Sci.* **12**, 1413–1425 (2019).

19. Z. Kachwalla, D. J. Miller, Transient spectroscopy using the Hall effect. *Appl. Phys. Lett.* **50**, 1438–1440 (1987).

20. A. Musiienko, F. Yang, T. W. Gries, C. Frasca, D. Friedrich, A. Al-Ashouri, E. Sağlamkaya, F. Lang, D. Kojda, Y.-T. Huang, V. Stacchini, R. L. Z. Hoye, M. Ahmadi, A. Kanak, A. Abate, Resolving electron and hole transport properties in semiconductor materials by constant light-induced magneto transport. *Nat. Commun.* **15**, 316 (2024).

21. O. Gunawan, S. R. Pae, D. M. Bishop, Y. Virgus, J. H. Noh, N. J. Jeon, Y. S. Lee, X. Shao, T. Todorov, D. B. Mitzi, B. Shin, Carrier-resolved photo-Hall effect. *Nature* **575**, 151–155 (2019).

22. O. Gunawan, Y. Virgus, K. Fai Tai, A parallel dipole line system. *Appl. Phys. Lett.* **106**, 062407 (2015).

23. O. Gunawan, T. Gokmen, Hall measurement system with rotary magnet, US patent 9,041,389 (2015).

24. S. M. Sze, K. K. Ng, "Ch. 1 Physics and properties of semiconductors – A review" in *Physics of Semiconductor Devices* (Wiley, ed. 3, 2006), pp. 34–35.

25. J. Euvrard, O. Gunawan, D. B. Mitzi, Impact of PbI_2 passivation and grain size engineering in $CH_3NH_3PbI_3$ solar absorbers as revealed by carrier-resolved photo-Hall technique. *Adv. Energy Mater. 9*, 1902706 (2019).

26. R. F. Pierret, G. W. Neudeck, "Ch. 4 Equilibrium carrier statistics" in *Advanced Semiconductor Fundamentals, vol. 6* (Addison-Wesley, ed. 2, 1987), p. 120.

27. A. Usami, T. Natori, A. Ito, S.-I. Ishigami, Y. Tokuda, T. Wada, Study of electrical properties of defects in SOI films by wafer bonding. *MRS Onl. Proc. Lib.* **262**, 349–354 (1992).

28. M. Kim, J. Jeong, H. Lu, T. K. Lee, F. T. Eickemeyer, Y. Liu, I. W. Choi, S. J. Choi, Y. Jo, H.-B. Kim, S.-I. Mo, Y.-K. Kim, H. Lee, N. G. An, S. Cho, W. R. Tress, S. M. Zakeeruddin, A. Hagfeldt, J. Y. Kim, M. Grätzel, D. S. Kim, Conformal quantum dot–SnO_2 layers as electron transporters for efficient perovskite solar cells. *Science* **375**, 302–306 (2022).

29. S. Ravishankar, T. Unold, T. Kirchartz, Comment on "Resolving spatial and energetic distributions of trap states in metal halide perovskite solar cells". *Science* **371**, eabd8014 (2021).

30. M. Kim, G. H. Kim, T. K. Lee, I. W. Choi, H. W. Choi, Y. Jo, Y. J. Yoon, J. W. Kim, J. Lee, D. Huh, H. Lee, S. K. Kwak, J. Y. Kim, D. S. Kim, Methylammonium chloride induces intermediate phase stabilization for efficient perovskite solar cells. *Joule* **3**, 2179–2192 (2019).

31. W. Shockley, W. Read Jr., Statistics of the recombinations of holes and electrons. *Phys. Rev.* **87**, 835–842 (1952).

32. R. N. Hall, Electron-hole recombination in germanium. *Phys. Rev.* **87**, 387–387 (1952).

33. R. A. Smith, "Ch. 5 Electron transport phenomena" in *Semiconductors* (Cambridge, ed. 2, 1978), p. 109.

34. I. Levine, S. Gupta, A. Bera, D. Ceratti, G. Hodes, D. Cahen, D. Guo, T. J. Savenije, J. Avila, H. J. Bolink, O. Millo, D. Azulay, I. Balberg, Can we use time-resolved measurements to get steady-state transport data for halide perovskites? *J. Appl. Phys.* **124**, 103103 (2018).

35. Matrix representation of conic sections. Wikipedia, https://en.wikipedia.org/wiki/Matrix_representation_of_conic_sections, accessed Oct 2024.

36. Conic section. Wikipedia, https://en.wikipedia.org/wiki/Conic_section, accessed Oct 2024.

37. A. B. Ayoub, The central conic sections revisited. *Math. Mag.* **66**, 322–325 (1993).

38. Hyperbola. Wikipedia, https://en.wikipedia.org/wiki/Hyperbola, accessed Oct 2024.

39. K. J. Meech, R. Weryk, M. Micheli, J. T. Kleyna, O. R. Hainaut, R. Jedicke, R. J. Wainscoat, K. C. Chambers, J. V. Keane, A. Petric, L. Denneau, E. Magnier, T. Berger, M. E. Huber, H. Flewelling, C. Waters, E. Schunova-Lilly, S. Chastel, A brief visit from a red and extremely elongated interstellar asteroid. *Nature* **552**, 378–381 (2017).

40. H. Yin, A. Akey, R. Jaramillo, Large and persistent photoconductivity due to hole-hole correlation in CdS. *Phys. Rev. Mater.* **2**, 084602 (2018).

41. D. Ompong, K. S. Ram, D. D. Y. Setsoafia, H. Mehdizadeh Rad, J. Singh, Saturation of open-circuit voltage at higher light intensity caused by interfacial defects and nonradiative recombination losses in perovskite solar cells. *Adv. Mater. Interfaces* **10**, 2201578 (2022).

42. P. Caprioglio, M. Stolterfoht, C. M. Wolff, T. Unold, B. Rech, S. Albrecht, D. Neher, On the relation between the open-circuit voltage and quasi-Fermi level splitting in efficient perovskite solar cells. *Adv. Energy Mater.* **9**, 1901631 (2019).

43. Y. Chen, H. T. Yi, X. Wu, R. Haroldson, Y. N. Gartstein, Y. I. Rodionov, K. S. Tikhonov, A. Zakhidov, X.-Y. Zhu, V. Podzorov, Extended carrier lifetimes and diffusion in hybrid perovskites revealed by Hall effect and photoconductivity measurements. *Nat. Commun.* **7**, 12253 (2016).

44. P. Caprioglio, C. M. Wolff, O. J. Sandberg, A. Armin, B. Rech, S. Albrecht, D. Neher, M. Stolterfoht, On the origin of the ideality factor in perovskite solar cells. *Adv. Energy Mater.* **10**, 2000502 (2020).

45. J. Nelson, "Ch. 4 Generation and recombination" in *The Physics of Solar Cells* (Imperial College, 2003), p. 79.

46. T. Kirchartz, U. Rau, What makes a good solar cell? *Adv. Energy Mater.* **8**, 1703385 (2018).

47. S. Wang, W. B. Xiao, F. Wang, Structural, electronic, and optical properties of cubic formamidinium lead iodide perovskite: A first-principles investigation. *RSC Adv.* **10**, 32364–32369 (2020).

48. R. H. Bube, "Ch. 2 Photoconductivity parameters" in *Photoelectronic Properties of Semiconductors* (Cambridge Univ., 1992), p.19.

49. S. Heo, G. Seo, Y. Lee, D. Lee, M. Seol, J. Lee, J. B. Park, K. Kim, D. J. Yun, Y. S. Kim, J. K. Shin, T. K. Ahn, M. K. Nazeeruddin, Deep level trapped defect analysis in $CH_3NH_3PbI_3$ perovskite solar cells by deep level transient spectroscopy. *Energ. Environ. Sci.* **10**, 1128–1133 (2017).

50. C. W. Warren, E. T. Roe, D. W. Miller, W. N. Shafarman, M. C. Lonergan, An improved method for determining carrier densities via drive level capacitance profiling. *Appl. Phys. Lett.* **110**, 203901 (2017).

51. M. A. Lampert, Volume-controlled current injection in insulators. *Rep. Prog. Phys.* **27**, 329–367 (1964).

52. V. M. Le Corre, E. A. Duijnstee, O. El Tambouli, J. M. Ball, H. J. Snaith, J. Lim, L. J. A. Koster, Revealing charge carrier mobility and defect densities in metal halide perovskites via space-charge-limited current measurements. *ACS Energy Lett.* **6**, 1087–1094 (2021).

53. R. R. Haering, E. N. Adams, Theory and application of thermally stimulated currents in photoconductors. *Phys. Rev.* **117**, 451–454 (1960).

54. A. Baumann, S. Väth, P. Rieder, M. C. Heiber, K. Tvingstedt, V. Dyakonov, Identification of trap states in perovskite solar cells. *J. Phys. Chem. Lett.* **6**, 2350–2354 (2015).

55. R. Brenes, D. Guo, A. Osherov, N. K. Noel, C. Eames, E. M. Hutter, S. K. Pathak, F. Niroui, R. H. Friend, M. Saiful Islam, H. J. Snaith, V. Bulović, T. J. Savenije, S. D. Stranks, Metal halide perovskite polycrystalline films exhibiting properties of single crystals. *Joule* **1**, 155–167 (2017).
